# Designing Electronic Problem-Solving Training for Individuals With Traumatic Brain Injury: Mixed Methods, Community-Based, Participatory Research Case Study

**DOI:** 10.2196/83995

**Published:** 2026-01-20

**Authors:** Matthew Schmidt, Yueqi Weng, Shannon Juengst, Alexandra Holland

**Affiliations:** 1 College of Pharmacy, Department of Clinical and Administrative Pharmacy, University of Georgia, River's Crossing, 215, Athens, GA, 30602, United States, 1 (706) 542-3000; 2 Department of Workforce Education and Instructional Technology, Mary Francis Early College of Education, University of Georgia, Athens, GA, United States; 3 Brain Injury Research Center, TIRR Memorial Hermann, Houston, TX, United States

**Keywords:** traumatic brain injury, community-based participatory research, user-centered design, usability, mHealth, rehabilitation, problem-solving training

## Abstract

**Background:**

Traditional rehabilitation research often excludes the voices of individuals with lived experience of traumatic brain injury (TBI), resulting in interventions that lack relevance, accessibility, and effectiveness. Community-based participatory research (CBPR) offers an alternative framework that emphasizes collaboration, power sharing, and sustained engagement with patients, caregivers, and clinicians.

**Objective:**

This study aimed to apply CBPR to guide front-end design (empathy interviews, empathy mapping, personas) and to evaluate the sociotechnical-pedagogical usability of the Electronic Problem-Solving Training (ePST) mobile health (mHealth) intervention with TBI partners.

**Methods:**

A multistep, mixed methods design case methodology was adopted, guided by CBPR principles and learning experience design. Participatory mechanisms included a 33-member Community Advisory Board and 10 Community Engagement Studios that engaged TBI survivors, caregivers, clinicians, and researchers throughout the Discover, Define, Develop, and Deliver phases of the Double Diamond model. Iterative activities included empathy interviews (n=14), persona development (n=10), rapid prototyping, and usability testing with 5 participants with TBI using think-aloud protocols and the Comprehensive Assessment of Usability for Learning Technologies instrument.

**Results:**

The co-design process successfully translated community feedback into an empathy-informed, user-centered prototype and systematically identified design considerations that single-partner approaches overlook. TBI-specific design requirements emerged, including the need for linear content progression over branching navigation, higher technical performance standards, and explicit content signaling with clarity prioritized over novel interface design. Think-aloud protocols revealed that participants struggled with mobile navigation and branching structures but excelled with sequential content progression. In addition, the input from individuals with TBI, caregivers, clinicians, and researchers led to practical refinements such as shorter microlearning lessons (5‐12 min), clearer voiceover tone, and simplified navigation, directly addressing the study’s objective of improving accessibility and emotional resonance. Overall usability was high, measured using the Comprehensive Assessment of Usability for Learning Technologies (CAUSLT), with an average score of 4.25 out of 5 (SD 0.72; 95% CI 3.36‐5.15; n=5). Knowledge accuracy was 80% (8/10 items; 95% CI 49%‐94%; n=5 participants; 2 items each), indicating that the system effectively supported learning and comprehension. Module completion was 100% (5/5; 95% CI 56.6%‐100%). Average time-on-task for 10 lesson completions was 11.47 (SD 5.28; range 4.6‐21.42) minutes per lesson, demonstrating strong task efficiency and engagement. Highest ratings were observed in the pedagogical usability domain, reflecting that the interface was clear, intuitive, and conducive to learning. Collectively, these findings suggest that applying CBPR across all design stages produced a technically sound, easy-to-use, and pedagogically meaningful mHealth tool specifically tailored for individuals with TBI.

**Conclusions:**

Sustained CBPR across full design and development cycles resulted in high usability for ePST for individuals with TBI. Ultimately, this study operationalized a full-cycle pipeline that links sustained community partnership to measured usability outcomes, producing community-informed design principles and a reproducible mixed methods approach for formative mHealth development for TBI.

## Introduction

### Traumatic Brain Injury Rehabilitation

Traditional rehabilitation research underrepresents people with lived experience of disability, including traumatic brain injury (TBI), yielding interventions misaligned with patient contexts [1-4]. TBI has acute and chronic sequelae [5,6] affecting cognition, emotion, and social functioning [5-10] that adversely affect learning and care access [11,12]. When design ignores these constraints, relevance and engagement drop [13]. Although recent studies demonstrated the feasibility of participatory adaptations in TBI rehabilitation [14,15], such approaches remain rare [16]. Emerging protocols increasingly incorporate caregiver and community voices through community-based participatory research (CBPR) frameworks [17], yet broader adoption remains limited [18]. Indeed, although chronic challenges faced by individuals with TBI are increasingly recognized, rehabilitation research rarely translates this awareness into meaningful community engagement or integration of practitioner perspectives [19,20]. Provider- and institution-centered models continue to dominate, reinforcing inequities [21] and limiting collaboration between researchers, clinicians, and patients [22,23]. This results in less responsive interventions, lower user satisfaction, and reduced effectiveness [5,24-26]. Despite growing support for participatory approaches, provider-centric norms persist [27]. This study responds to those gaps by modeling a collaborative, community-informed design process [18,28].

Power sharing and collaborative decision-making are critical to designing effective, context-responsive interventions [28,29]. Rehabilitation requires real-world interaction, collaboration, and adaptability to individual needs [30]. Participatory approaches shift decision-making toward community members [31], having produced measurable improvements in health outcomes and patient-reported measures [32]. For example, ethnographic work by Manhas and colleagues [33] showed shared decision-making in rehabilitation enhances patient satisfaction, understanding, goal attainment, and self-reported outcomes. This contrasts with provider-driven models that limit patient involvement and flexibility. Indeed, challenges such as limited community engagement, asymmetrical decision-making, and provider-centered research can undermine the relevance and impact of TBI rehabilitation efforts [19,21-23]. These issues call for more inclusive approaches that prioritize symmetrical decision-making and meaningful collaboration with the TBI community [33].

This paper presents a case example of the formative design and evaluation of Electronic Problem-Solving Training (ePST), a metacognitive, evidence-based mobile health (mHealth) problem-solving intervention. ePST is based on PST, a cognitive-behavioral approach with proven efficacy for neurodevelopmental and psychological conditions that is grounded in some of the strongest evidence in cognitive rehabilitation [34,35]. PST and comparable approaches are widely used in psychology to improve problem-solving skills and mindset [36,37] and have shown promise for preventing and treating cognitive deficits [38] through numerous clinical trials [39]. Research suggests PST can be especially beneficial for long-term or multifaceted health issues, such as TBI [40,41]. A robust body of evidence shows that such problem-solving approaches lead to meaningful reductions in symptoms, strengthen individuals’ confidence in managing their health, and enhance adherence to prescribed regimens [42-44]. ePST was developed using learning experience design and a CBPR framework to ensure accessibility, community-driven decision-making, and iterative co-design [18,45,46]. Learning experience design and CBPR guided front-end activities and the sociotechnical-pedagogical usability evaluation reported here.

### Background and Rationale

Rehabilitation research often centers around clinician and designer perspectives over patient input, reducing relevance, effectiveness, and adaptability for individuals with TBI [47,48]. Correa and colleagues [49] showed that interventions lacking patient involvement can be misaligned with how patients perceive risks, benefits, and treatment goals, undermining recruitment and randomization. Such problems suggest a need for adaptive, patient-informed approaches, which CBPR can provide in a context-sensitive and ethical manner [46]. CBPR helps researchers understand lived experience and co-create interventions that are more relevant, acceptable, and effective. For example, Quilico and colleagues [28] partnered with people with TBI and caregivers to adapt a physical activity program, producing changes that improved relevance, outcomes, and engagement. Groussard and colleagues [50] involved users with lived TBI experience and caregivers in developing and evaluating a cognitive support system, yielding improved user satisfaction and greater autonomy. However, participation alone is insufficient. CBPR requires reciprocal relationships among community members, academics, and practice partners to draw on diverse strengths [51]. As a case-in-point, Springer and Skolarus [52] specifically distinguished between the “community-based” and “participatory” components of CBPR to clarify how all components of this approach are needed to promote sustained, power-sharing partnerships.

As CBPR is applied increasingly to digital health interventions like ePST, new design and evaluation demands emerge. For example, reporting in CBPR remains inconsistent, and implementation is uneven, especially in rehabilitation contexts [16]. Usability and contextual fit present persistent barriers to adoption in eHealth and mHealth, reinforcing the need for community-informed design and iterative testing cycles [53]. In addition, promoting sustained engagement remains a challenge [18,54], which supports the use of innovative pedagogical strategies such as microlearning, a design approach shown to improve engagement and learning outcomes in health applications when lessons are limited to a length of 5 minutes to 12 minutes [55]. In parallel, sociotechnical frameworks have been recommended for evaluating patient-facing tools, supporting our integration of CBPR, learning experience design, and the sociotechnical-pedagogical framework (discussed in the next section) [56]. Collectively, these gaps suggest a need to balance TBI rehabilitation complexity with the provision of usable, accessible, and engaging interventions. We illustrate our approach to achieving this balance through the conceptual framework we present in the following section.

### Conceptual Framework

**Figure 1.**
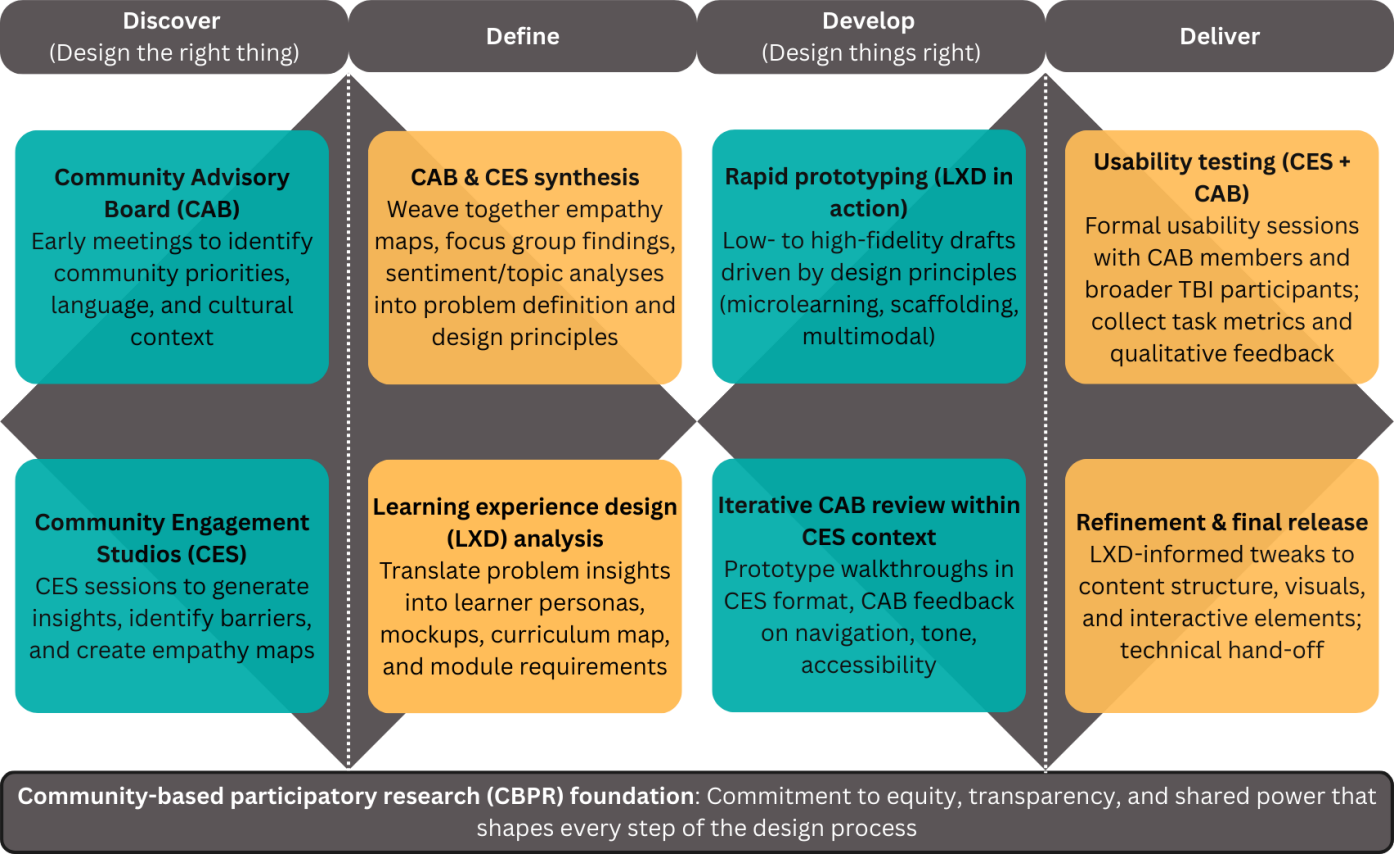
Conceptual framework integrating community-based participatory research, the Community Advisory Board (CAB), Community Engagement Studios (CES), and learning experience design (LXD) mapped onto the Double Diamond design framework for individuals with traumatic brain injury (TBI).

We developed a conceptual model that places CBPR at the methodological core, pairs participatory mechanisms (Community Advisory Board or Community Engagement Studios) with learning experience design to convert community partner input into design principles, and maps these strands onto the Double Diamond (Figure 1) for iterative development and sociotechnical-pedagogical evaluation [57-61].

#### CBPR as the Foundational Ethos

We adopted CBPR as a foundational ethos to foster inclusive, patient-centered rehabilitation design and to translate community priorities into practice [62-65]. Central to CBPR are collaboration and balanced partnerships that share decision-making responsibility [66]. Unlike short-term, investigator-led studies, CBPR emphasizes long-term reciprocal relationships that promote ethical research practices and improved outcomes [67]. This is important because top-down, limited-duration studies can erode trust and exclude local needs, with standardized practices that do not accommodate community input tending to perpetuate these problems [68,69]. CBPR’s emphasis on shared decision-making across all phases of the research process provides one avenue to address these problems [70]. Collaboration through structured partnerships allows community members to inform priorities, participate in knowledge creation, and strengthen the real-world applicability of interventions [31]. These approaches move research beyond expert-driven agendas by integrating the lived experiences, priorities, and contextual knowledge of community members into the design and implementation process [71].

#### Participatory Mechanisms

##### Community Advisory Board

Community Advisory Boards are structured, ongoing partnerships that integrate people with lived experience into research, providing authentic representation and culturally grounded input across the project lifecycle [72-75]. Unlike short-term focus groups, Community Advisory Boards meet regularly to co-develop research strategy, advise on ethics and context, and guide intervention refinement, fostering shared leadership, trust, and power sharing [76-79].

##### Community Engagement Studios

Community Engagement Studios are structured, facilitated consultations in which researchers obtain targeted feedback from panels of community experts, caregivers, and clinicians. Unlike advisory boards or focus groups, Community Engagement Studios use focused, iterative sessions to promote dialogue, reciprocal learning, and sustained community involvement [59,80]. Originating with the Meharry-Vanderbilt Community-Engaged Research Core [58], Community Engagement Studios were developed to overcome participation barriers in clinical and rehabilitation research, including mistrust from historical unethical practices and social inequities [81]. By positioning community members as consultants and experts rather than passive subjects, Community Engagement Studios help identify barriers, adapt interventions to community needs, and build trust with underrepresented groups [82,83]. Community Engagement Studios can enhance cultural adaptation [84], increase minority participation [82,83], and reduce power imbalances between researchers and community members [85,86].

### Operationalization via Learning Experience Design

Learning experience design is a learner-centered, theoretically grounded framework that integrates instructional design, cognitive science, user experience, and participatory approaches [87,88]. Learning experience design emphasizes designing engaging and inclusive learning environments that respond to learners’ real-world needs and experience [89,90]. Learning experience design focuses on the cognitive, emotional, and perceptual influences of learner interactions with content, tools, and people across the learning process [91-93]. Learning experience design guided ePST’s Double Diamond workflow. In *Discover*, empathy interviews identified core needs and constraints; in *Define*, those insights plus Community Advisory Board input shaped personas, module structure, and mock content [94,95]. To address TBI-specific cognitive limits (eg, memory, fatigue), the *Develop* phase adopted microlearning (ie, short, digestible lessons lasting 5 minutes to 12 minutes) intended to lower cognitive load, promote encoding, and support retention [96-99]. *Deliver* used iterative usability testing to validate designs and drive refinements. Multimodal strategies (text, visuals, voiceover, interactivity) and gamification (badges, progress indicators, interactive tasks) supported diverse preferences and motivation [100,101].

#### Sociotechnical-Pedagogical Framework

The sociotechnical-pedagogical framework conceptualizes learner experience as the alignment of 3 interdependent domains: technological, pedagogical, and sociocultural [102,103]. The technological domain covers reliability, accessibility, device compatibility, navigability, and error tolerance; the pedagogical domain covers alignment of objectives, materials, activities, and assessment, plus clarity, scaffolding, cognitive load management, and feedback quality; and the sociocultural domain addresses presence, identity, communication, cultural responsiveness, and scenario authenticity. The sociotechnical-pedagogical framework serves as both a design and evaluation lens, operationalized via dimension-specific heuristics validated against course evaluations that identified 195 distinct problems consolidated into nonoverlapping heuristics spanning the 3 domains [102]. This approach is critical in neurorehabilitation because traditional usability frameworks often miss interactions among cognitive, social, and technical factors [88]. For people with TBI, technological design must go beyond basic accessibility to reduce cognitive load (simplified interfaces, memory supports, fatigue accommodations) and ensure assistive-technology compatibility. Pedagogical design should address executive function limits via clear structure, predictable flows, compensatory strategies, repetition, and metacognitive scaffolds to support transfer. Sociocultural design must attend to stigma, identity shifts after injury, peer and family involvement, and social-context fit. The sociotechnical-pedagogical framework reveals problems that purely technical reviews miss.

#### Intervention Description

**Figure 2.**
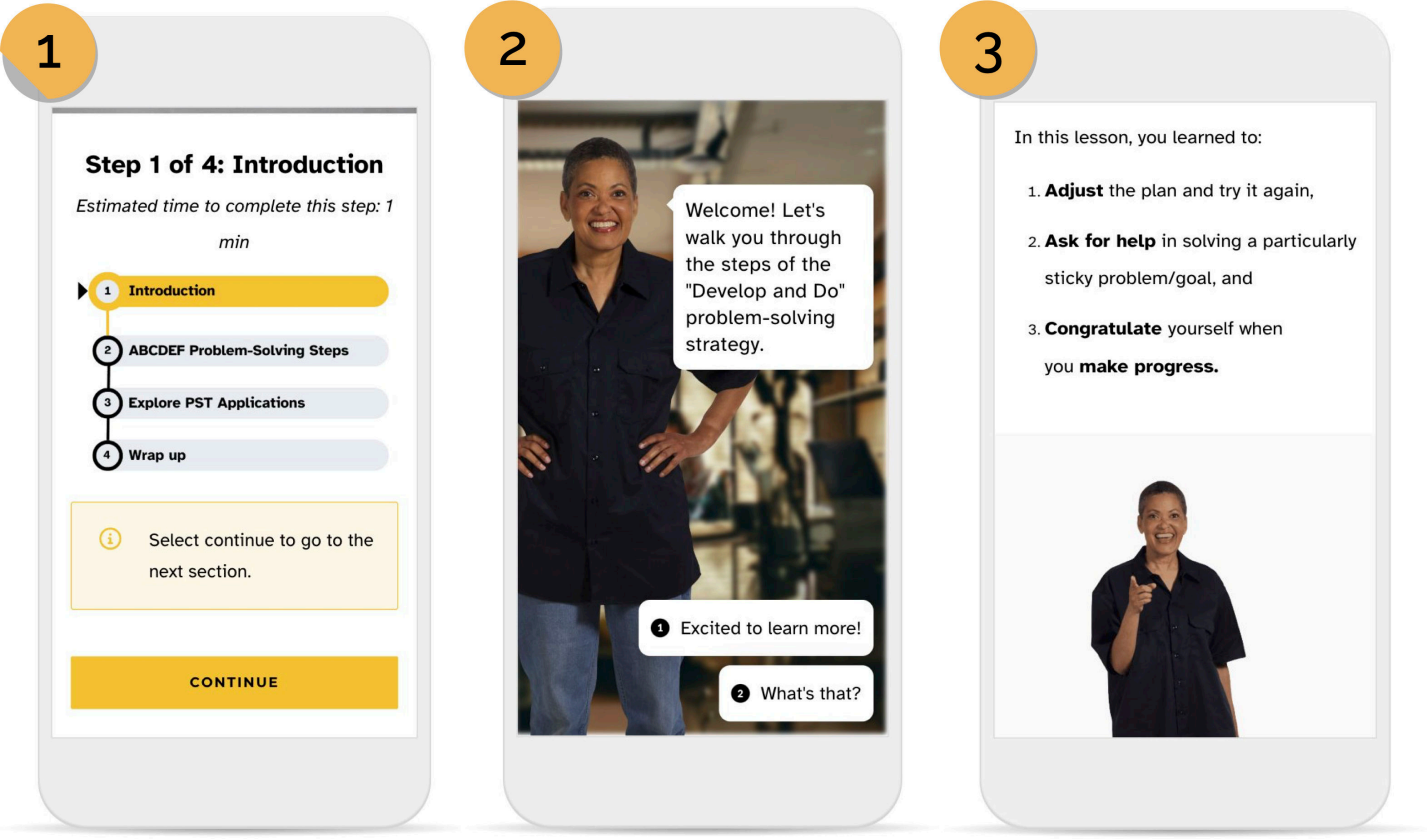
Representative screenshots from the Electronic Problem-Solving Training (ePST) prototype and final user interface, captured during usability testing with adults with traumatic brain injury: (1) progress tracker, (2) virtual coach “Ruth” interface, and (3) reminder/notification panel.

**Figure 3.**
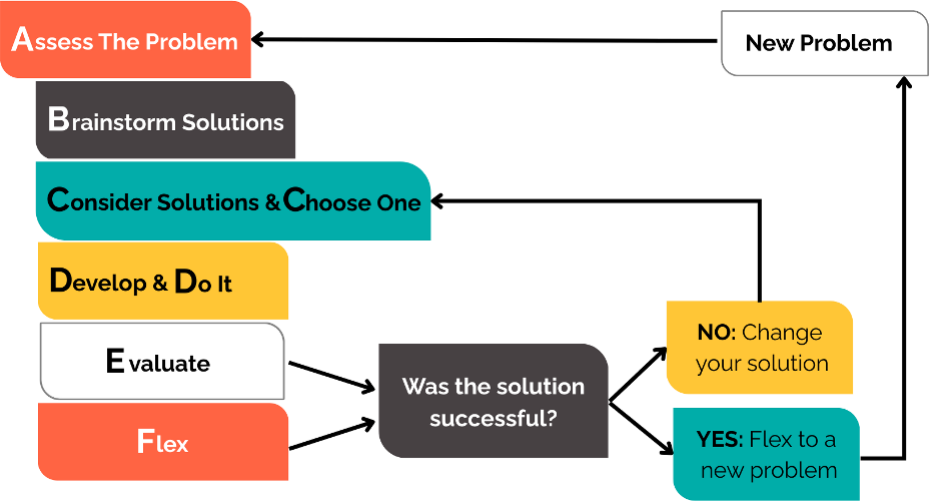
Problem-solving training strategy diagram showing the ABCDEF 6-step metacognitive process implemented in Electronic Problem-Solving Training (ePST): assess, brainstorm, consider and choose, develop and do, evaluate, and flex.

ePST is a cross-platform, community-informed mHealth intervention tailored to the cognitive and emotional needs of adults with TBI. Built on microlearning, it delivers short (5‐12 minutes), chunked lessons with built-in progress tracking to reduce cognitive load. Engagement features include motivational messaging derived from empathy interviews; a virtual coach (“Ruth”); personalized learning pathways; embedded reminders; and gamified elements (badges, certificates) to support memory, reinforce learning, and sustain motivation (Figure 2). ePST is grounded in problem-solving training and operationalizes the 6-step ABCDEF mnemonic (Figure 3): A, assess the problem; B, brainstorm solutions; C, consider and choose; D, develop and do; E, evaluate; and F, flex. ePST translates these steps into scaffolded modules that teach structured decision-making and problem-solving strategies tailored to adults with TBI. A description of the ePST learning modules is provided in Multimedia Appendix 1.

### Purpose and Research Questions

The purpose of this iterative, multimethod formative design and evaluation case study was to use a CBPR approach to guide front-end design activities (ie, empathy interviews, empathy mapping, and persona development) and to evaluate the sociotechnical-pedagogical usability of the ePST intervention with TBI community members (ie, Community Advisory Board members, families, providers, and individuals with lived TBI experience) at a large public university and a large medical center in the southern United States. The questions that guided this research included: research question (RQ) 1: What themes related to learning needs, barriers, and preferences emerge from front-end design activities (empathy interviews, empathy mapping, and persona development) with TBI community members? RQ 2: How did individuals with TBI perceive the sociocultural, technological, and pedagogical usability aspects of their experience with ePST during testing? RQ 3: How were identified sociotechnical-pedagogical usability issues addressed through design refinements?

## Methods

### Double Diamond Approach

This multimethod formative design and evaluation study followed the Double Diamond approach (Discover, Define, Develop, Deliver) and ran from February 2024 through July 2024. In *Discover*, we established the Community Advisory Board, drafted initial design principles, and conducted Community Engagement Studio empathy interviews with people with TBI. In *Define*, we developed learner personas, produced a curriculum map, and iteratively refined priorities via Community Engagement Studios and Community Advisory Board reviews. In *Develop*, we translated principles into low- to high-fidelity prototypes and internal subject matter expert review. In *Deliver*, we conducted iterative usability testing with people with lived TBI experience and implemented refinements after each round. We reported patient and public involvement using the GRIPP2 Short Form (GRIPP2-SF) [104]. A 1-page mapping table linking GRIPP2-SF items to manuscript locations is provided in Multimedia Appendix 1.

### Participants

#### Community Advisory Board Participants

The Community Advisory Board (n=33) was purposively assembled to include people with lived TBI experience, caregivers, clinicians, researchers, industry representatives, advocates, and members of minoritized groups. Members were identified via professional networks, partner clinics, and community organizations and invited by email. Selection criteria were TBI or digital health expertise, lived experience, demographic diversity, and advocacy and service representation. Community Advisory Board members received US $25 per meeting. Community Advisory Board composition is provided in Multimedia Appendix 1.

#### Empathy Interview Participants

Empathy interview participants (n=14) were recruited via clinician referral and community outreach at a large tertiary rehabilitation center in the southern United States in February 2024 and March 2024. Inclusion criteria were age ≥18 years, proficiency in English, and either (1) documented TBI confirmed by clinician referral or review of medical records when available or (2) self-reported TBI with screening confirmation of capacity to participate. Exclusion criteria were severe communication impairments or acute medical instability that precluded informed consent or participation. Caregivers and providers were eligible if they provided regular care or clinical services to adults with TBI.

#### Usability Testing Participants

Usability testing participants (n=5) were recruited purposively from the same clinical and community sources in July 2024 to capture variation in technology experience and time since injury. Inclusion criteria included age ≥18 years, English fluency, history of TBI (clinician referral or medical record when available), ability to use a smartphone or computer without assistance, and capacity to provide informed consent and follow study tasks. Exclusion criteria included acute medical or psychiatric instability and severe receptive or expressive communication impairments that prevented participation. Five participants is standard for early-stage formative usability tests, with 80% of usability problems identified via small samples [105,106]. This low number limits statistical generalizability but is conventional in heuristic-based usability work intended for problem identification [107,108]. This approach has substantial precedent in digital health formative studies that use small, purposive usability samples to drive iterative refinements [109-111]. To increase rigor and reduce bias from the small sample, we purposively sampled, triangulated findings, and applied an iterative refine-and-retest logic.

### Ethical Considerations

The study protocol was approved by the Human Research Protection Program at the University of Georgia (IRB #00009943) on June 17, 2024, and was deemed exempt. Written informed consent was obtained electronically via Qualtrics. Participants reviewed the full consent document, typed their full name and the date to indicate agreement, and submitted the consent form. The consent form covered study purpose and procedures, audio and video recording, foreseeable risks and benefits, right to withdraw, compensation, and data handling. All study data were de-identified and stored on encrypted University of Georgia servers with access limited to authorized study personnel. The master linking list and raw recordings will be destroyed at study end; de-identified data may be used for future research but will not be deposited in a public repository. Participants received US $25 per activity, and Community Advisory Board members were paid US $25 per meeting, with payments issued via Clincard after each session. Payments were institutional review board–approved and described in the consent forms. No identifying information was included in this paper or multimedia appendices.

### Procedures

Given the iterative nature of the Double Diamond approach, analysis was multimodal and occurred across all phases of design, with analysis falling into 3 broad categories: (1) qualitative, (2) quantitative, and (3) computational. No data were missing for any of the reported analyses.

#### Discover Phase Procedures

##### Establishment of the Community Advisory Board

Community Advisory Board members were recruited purposively [112] from the community, academia, industry, and medical-related institutes based on 4 criteria: (1) professional expertise in TBI rehabilitation, assistive technology, and or digital health; (2) lived experience with TBI; (3) demographic diversity across age, gender, socioeconomic status, and geography; and (4) community member representation including those with lived experience, clinicians, researchers, technology developers, and advocacy organizations. Quarterly Community Advisory Board meetings were held across Phases 1‐3 (total n=16).

##### Establishment of Preliminary Design Principles

We drew on findings from a prior study, *Caregivers in Dementia PST and DSJ* (CaDeS), which tested coach-delivered PST [113]. Open-ended responses to overall intervention satisfaction were analyzed using machine learning techniques, including sentiment analysis and latent Dirichlet allocation, to generate an initial set of 7 design principles for ePST, which were reviewed and refined in a subsequent Community Engagement Studio session.

##### Empathy Interviews

Empathy interviews were conducted with 3 groups of participants. Groups 1 (n=6) and 2 (n=3) consisted of TBI survivors. Group 3 consisted of care partners and providers (n=5). Interviews were guided by the 4-phase empathy framework from Kouprie and Visser [114]. All interviews were approximately 75 minutes and conducted online using Zoom web conferencing software. Questions focused on (1) learning challenges, (2) effective therapies, (3) the impact of others’ stories, (4) group-specific challenges, (5) building trust through shared expertise, and (6) motivational messages. Interviews were recorded and transcribed using Zoom.

### Define Phase Procedures

#### Empathy Mapping

Empathy mapping guided learner analysis and informed the design of ePST [115]. Empathy mapping involved synthesizing participants’ responses into 4 core domains (“Says,” “Thinks,” “Does,” and “Feels”) to foster understanding of their motivations, challenges, and learning preferences. This allowed capture of nuanced information about participants’ cognitive, emotional, and behavioral experiences. A total of 9 empathy maps were created (see Multimedia Appendix 1). These were then used to generate learner personas and referenced to inform design.

#### Persona Development

Personas are fictional, data-informed archetypes that represent individuals within the target population [116]. Our personas provided summaries of representative descriptors based on information that was synthesized from empathy maps. Personas were presented to the Community Advisory Board, reviewed, and revised. Initial designs included TBI severity; however, this was removed at the recommendation of the Community Advisory Board, as severity was an inadequate method to represent nuanced TBI characteristics, especially chronically. The final set of personas (n=10) is provided in Multimedia Appendix 1.

#### Refinement of Design Principles

Design principles were refined based on a structured empathy interview with 5 caregivers and providers. Analysis comprised a discussion-based analytic process to identify key insights from the transcripts. The design principles were then reviewed in a Community Engagement Studio session with the Community Advisory Board, who provided feedback on clarity, relevance, and completeness. Analysis did not focus on achieving saturation but instead prioritized triangulation across data sources and methods for development of design principles.

### Develop Phase Procedures

#### Community Engagement Studios

Structured Community Engagement Studio sessions were used to elicit structured feedback during Community Advisory Board meetings. Community Engagement Studio sessions (n=10) focused on usability challenges, content clarity, and delivery preferences. Community Engagement Studio sessions were between 60 minutes and 90 minutes, included 6 to 8 participants, and followed a structured protocol. A trained moderator guided discussion. Discussion foci varied depending on which design artifacts were being reviewed, Participants reflected on design artifacts’ clarity, relevance, and usability. Sessions were conducted, audio recorded, and transcribed using Zoom. Transcripts and notes were then synthesized into actionable design recommendations.

#### Rapid Prototyping

Rapid prototyping is an iterative design approach that quickly develops and refines working models based on user feedback [117]. This approach was used to transform insights from the *Define* phase into working prototypes. Initial design concepts were explored through low-fidelity mockups then iteratively refined into medium- and high-fidelity prototypes, with emphasis on flexibility and responsiveness to user input [117]. Designs were regularly reviewed during Community Engagement Studios for issues such as navigation, language complexity, and content pacing.

### Deliver Phase Procedures

Usability tests (n=5) were conducted by a trained graduate student and a university professor usability expert. Testing followed a semistructured, task-based research protocol. Sessions were between 60 minutes and 75 minutes and were conducted, recorded, and transcribed using Zoom. Participants completed 5 structured usability tasks per session while thinking aloud and sharing their screens. Tasks assessed both technological usability (eg, navigation, multimedia interaction) and pedagogical usability (eg, clarity of content, microlearning structure). Participants then completed the Comprehensive Assessment of Usability for Learning Technologies (CAUSLT) instrument [118]. Data were analyzed using an integrated approach that combined observational, survey, and efficiency metrics. Think-aloud transcripts and observer notes were reviewed and discussed by two team members to identify barriers. Responses to the CAUSLT instrument were summarized using descriptive statistics and disaggregated across the 3 instrument factors. Design flaws were prioritized using Nielsen’s severity scale [119]. Efficiency data were extracted from session recordings. Findings were documented in a report that was reviewed with the Community Advisory Board, whose feedback guided refinements in areas such as voiceover quality, mobile navigation, and content clarity for cognitive accessibility.

## Results

### Discover Phase Results

#### Composition of Community Advisory Board

The composition of the Community Advisory Board is presented in Table 1. Some individuals were represented in more than one group, as some Community Advisory Board members self-identified with more than one category.

**Table 1.** Composition of the Community Advisory Board (n=33) including counts and role categories for members recruited purposively from clinical partners, community organizations, academic networks, industry, and advocacy groups.

Category	Composition	Total representatives, n
Academic researchers	PhD researchers (n=12), psychologists (n=8), educational technology experts (n=2), graduate students (n=2)	24
Industry professionals	Software developer (n=1), software designers (n=5)	6
Individuals with lived experience and advocates	Individuals with TBI^a^ (n=6), TBI care partners (n=4), disability advocates (n=6)	16
Rehabilitation and clinical professionals	Occupational therapists (n=4), social workers (n=3), rehabilitation counselors (n=3)	10
Individuals with physical disabilities	Blind (n=1), deaf (n=1)	2
Individuals from minoritized groups	LGBTQAI+^b^ (n=4), minoritized racial and ethnic groups (n=6)	10

a TBI: traumatic brain injury.

b LGBTQAI+: lesbian, gay, bisexual, transgender, queer (or questioning), asexual (or allied), intersex, plus

#### Preliminary Design Principles

Preliminary design principles were established based on results of prior research and Community Advisory Board input. Principles emphasized accessibility, emotional resonance, clarity of messaging, and personalization, serving as our foundation for early prototypes, visual design, and engagement strategies. These preliminary principles were later expanded and structured into a comprehensive hierarchy, reported in the Define Phase Results section.

#### Empathy Interview Participant Demographics

We recruited 14 participants (Table 2) for empathy interviews, including individuals with TBI (n=9) and caregivers and providers (n=5).

**Table 2.** Participant demographics for empathy interviews (n=14), including de-identified breakdown by participant group, race or ethnicity, age bands, and gender.

Characteristics	Individuals with TBI^a^, n	Caregivers and providers, n
Race		
Hispanic	1	1
Caucasian or White	5	3
African American	2	0
Asian	0	1
Age (years)		
30-39	4	0
40-49	3	1
50-59	1	0
60-69	0	3
≥70	0	1
Gender		
Female	7	5
Male	2	0

a TBI: traumatic brain injury.

### Define Phase Results

#### Empathy Maps

Empathy maps were created (n=9), with each map including brief descriptors in the categories “Says,” “Thinks,” “Feels,” and “Does.” Analysis revealed 4 key themes characterizing the post-TBI experience: Participants experienced (1) frustration and disorientation with everyday tasks, (2) loss of self-identity and nostalgia for pre-injury life, (3) physical exhaustion from therapy that decreased motivation, and (4) social isolation due to perceived lack of family understanding. In response, participants developed adaptive strategies including structured skill relearning through rehabilitation and memory aids such as sticky notes. The full set of empathy maps is provided in Multimedia Appendix 1.

#### Personas

A set of personas (n=10) was created to guide design. Personas highlighted varied life contexts, recovery journeys, and learning needs across individuals such as veterans, students, professionals, and retirees. Each reflected unique combinations of cognitive, emotional, and physical challenges, along with personal goals like regaining independence, improving memory, or reducing stigma. Common facilitators included family support, adaptive tools, storytelling, and professional guidance. Despite varied barriers ranging from aphasia to fatigue to discrimination, all personas demonstrated resilience and motivation to recover. The complete set of personas is provided in Multimedia Appendix 1.

#### Refined Design Principles

A refined set of design principles was created in the *Define* phase, incorporating the preliminary set created during the *Discover* phase. Using a framework proposed by Kali [120], the design team organized these insights into a 3-tiered hierarchy (specific, pragmatic, and metaprinciples). Pragmatic principles reflected actionable guidance relevant to the learning design. These pragmatic principles were grouped into 6 broader metaprinciples, such as accessibility, emotional support, motivation, personalization, cultural relevance, and evidence-based action. Where applicable, specific principles (eg, interface features, content structures) were also identified to illustrate how the pragmatic principles would translate into concrete design decisions (Table 3).

**Table 3.** Design principles for Electronic Problem-Solving Training (ePST) module development, including metaprinciples, pragmatic principles, and specific principles derived from empathy interviews, empathy maps, Community Advisory Board and Community Engagement Studio feedback, and persona development.

Metaprinciple	Pragmatic principle	Specific principles
1. Ensure accessibility and usability	Design for cognitive and physical inclusion	Use clear, concise, jargon-free language; include closed captioning; support mobile-first navigation; design intuitive interaction patterns
1. Ensure accessibility and usability	Support memory and comprehension	Reinforce key concepts with reminders and visual anchors; use chunked content and repeated exposure
1. Ensure accessibility and usability	Allow flexible engagement	Enable learners to proceed at their own pace; allow pausing and resuming lessons easily
2. Support emotional and behavioral needs	Encourage emotional regulation	Include calming activities (eg, music, mindfulness cues); normalize behavioral variability in content
2. Support emotional and behavioral needs	Empathize with behavioral and communication challenges	Acknowledge and adapt for speech and behavioral limitations; use neutral, nonjudgmental tone
2. Support emotional and behavioral needs	Promote self-awareness and acceptance	Include prompts or reflection activities to build insight into strengths and limitations
3. Foster motivation and engagement	Use positive reinforcement	Integrate badges, rewards, and affirming feedback
3. Foster motivation and engagement	Emphasize goal setting and achievement	Provide explicit opportunities to set and track goals
3. Foster motivation and engagement	Provide regular feedback	Visual progress indicators; summary pages at lesson or module completion
4. Enable personalized and multimodal learning	Use varied sensory inputs	Combine visuals, audio narration, and interactivity
4. Enable personalized and multimodal learning	Allow for autonomy and independence	Design lessons that can be completed without facilitator support; scaffold progressively to reduce reliance on help
4. Enable personalized and multimodal learning	Tailor content for diverse learners	Include customizable avatars or pathways; vary representation and examples by demographic relevance
5. Establish credibility and cultural relevance	Include lived experience	Use testimonials from TBI^a^ survivors and care partners; embed quotes and real-world scenarios
5. Establish credibility and cultural relevance	Partner with trusted organizations	Reference TIRR^b^, advocacy groups, and clinical partners in content
5. Establish credibility and cultural relevance	Practice inclusive and representative design	Include diverse racial, ethnic, and gender identities; adapt content for veterans and other priority subgroups
6. Ground content in evidence and action	Communicate evidence accessibly	Present supporting research in simplified language or visuals; avoid academic jargon
6. Ground content in evidence and action	Use motivating calls to action	End modules with clear next steps (eg, “Enroll,” “Learn more”); include clickable links or guided follow-ups

a TBI: traumatic brain injury.

b TIRR:The Institute for Rehabilitation and Research.

### Develop Phase Results

**Figure 4.**
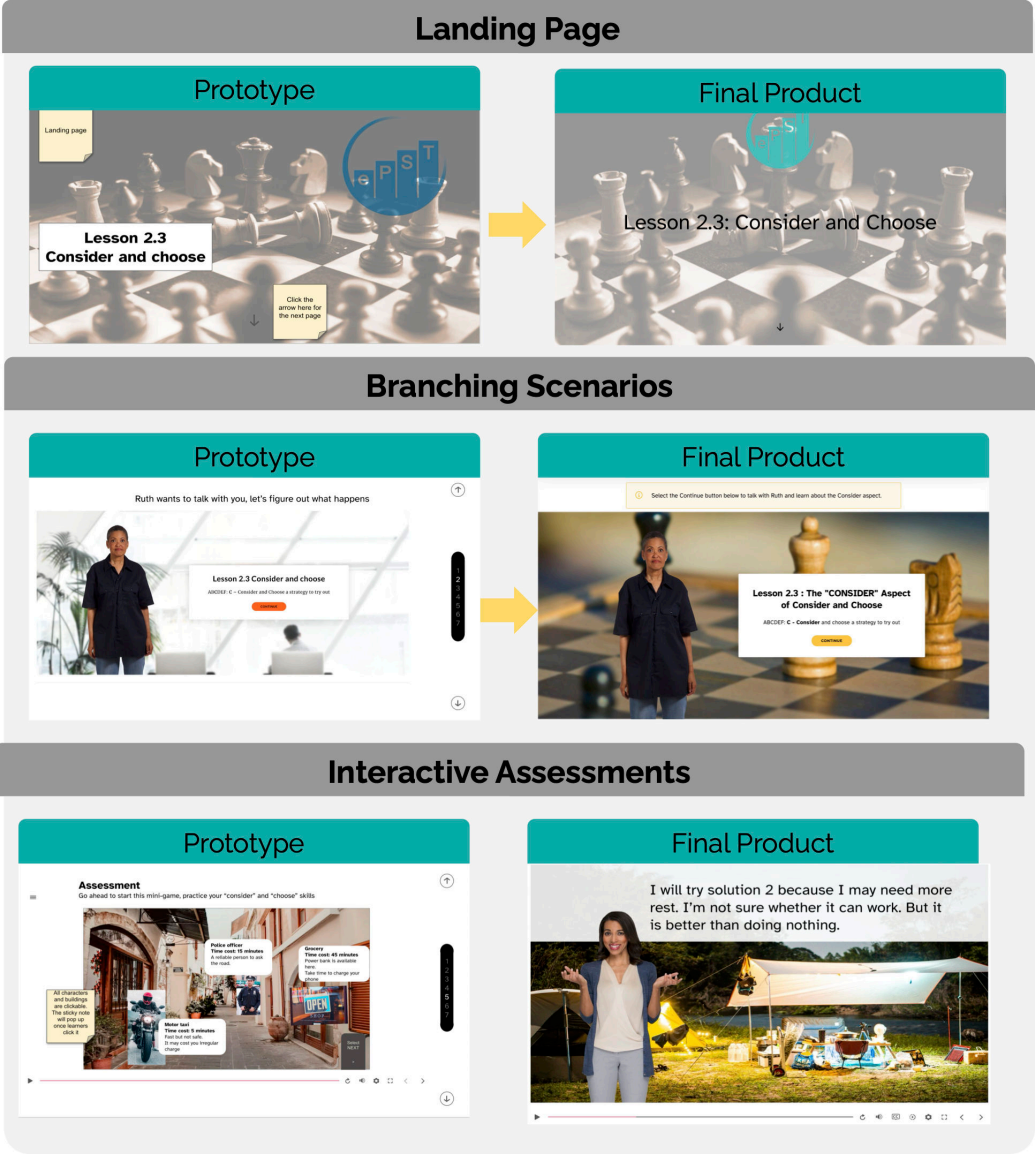
Evolution of selected Electronic Problem-Solving Training (ePST) design elements from prototypes to final product with panels illustrating iterative, prioritized changes driven by Community Advisory Board, Community Engagement Studios, and usability feedback (eg, badge redesign, microlearning length, voiceover tone, navigation simplification).

During the *Develop* phase, design artifacts progressed from low-fidelity storyboards to high-fidelity interactive prototypes (Figure 4). Low-fidelity mockups were iteratively refined into functional prototypes via structured Community Advisory Board feedback focused on usability, content clarity, accessibility, and delivery preferences. Key outputs included finalized lesson content, assessments, a cohesive visual design system, and functional prototypes. Community Engagement Studio sessions generated actionable recommendations that were synthesized into successive prototype iterations.

### Deliver Phase Results

#### Deliver Phase Participant Demographics

Participant demographics for the usability study are presented in Table 4.

**Table 4.** Usability study participant demographics (n=5) including individual-level characteristics of age, gender, race or ethnicity, education, employment, years since injury, and baseline technology experience.

Participant^a^	Age (years)	Gender	Race or ethnicity	Education	Employment status	Time since injury (years)	Technology experience
Leo	47	Male	Hispanic	High school	Permanent disability	17	Capable user, no eHealth experience
Morgan	31	Female	White	Some college	Stay-at-home spouse	11	Capable user, occasional eHealth use
Alexis	50	Female	Black	Bachelor’s degree	Permanent disability	20	Experienced user, frequent eHealth use
Riley	47	Male	White	Some college	Permanent disability	14	Experienced user, occasional eHealth use
Emma	36	Female	White	Some college	Stay-at-home parent	13	Experienced user, occasional eHealth use

a Participant names are pseudonyms.

#### Performance Metrics

All participants (n=5) finished every module (95% CI 56.6%‐100%). Lessons were completed efficiently, with participants spending about an average of 11.5 (SD 5.3; range 4.6‐21.4) minutes for 10 lesson completions. Knowledge checks showed solid comprehension (8/10 items correct; 95% CI 49%‐94%; n=5, 2 items each), meeting our objectives for task efficiency and learning support. Performance metrics are summarized in Table 5.

**Table 5.** Usability performance results and knowledge assessment, including task and efficiency measures (lesson completion time, tasks per lesson, task completion rate) and knowledge-item accuracy derived from recorded usability sessions (n=5).

Metric	Result
Efficiency measures	
Lesson completion time (minutes), mean (SD)	11.47 (5.28)
Completion time - Module 2 (minutes), mean	10.50
Completion time - Module 3 (minutes), mean	13.10
Time (minutes), range	4.6‐21.42
Tasks per user, mean	22.8
Tasks per lesson, mean	11.4
Task completion rate (tasks per minute), mean	0.996
Knowledge assessment	
Overall accuracy (% correct)	80
Question 1 accuracy (% correct)	60
Question 2 accuracy (% correct)	100

### CAUSLT Usability Assessment

**Figure 5.**
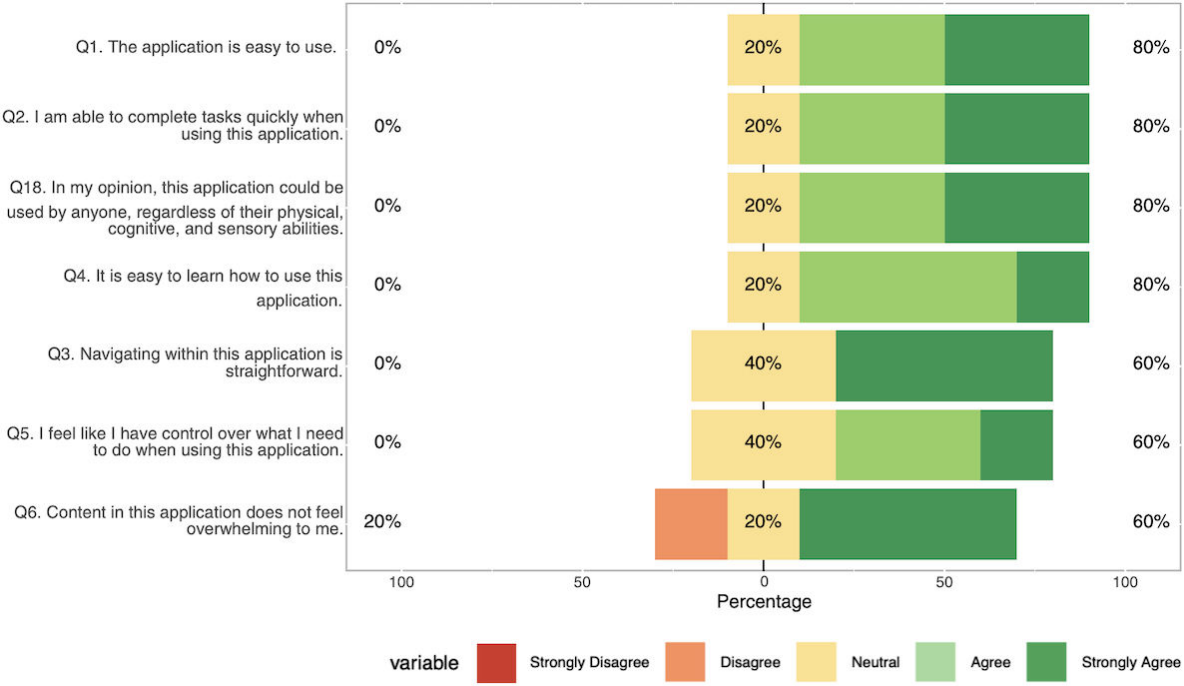
Technological usability responses collected during usability testing using the Comprehensive Assessment of Usability for Learning Technologies (CAUSLT), showing domain and item-level means and SDs for the technological domain (navigation, performance, error tolerance).

Participants completed the CAUSLT, which evaluates 3 dimensions of usability in educational technology using a 5-point Likert scale (1=Strongly Disagree, 5=Strongly Agree). Overall usability was high on the CAUSLT, with a mean score of 4.25 out of 5 (SD 0.72; 95% CI 3.36‐5.15; n=5), supporting our objective that the prototype be easy to use and learn. Results are presented in Table 6 and illustrated in Figures5^7^ .

**Table 6.** Usability scores by sociotechnical-pedagogical domain for technological, pedagogical, and sociocultural usability as measured using the Comprehensive Assessment of Usability for Learning Technologies (CAUSLT).

Usability dimension	Score, mean (SD)		Score, range	
Technological usability	4.06 (0.95)		3.50-5.00	
Pedagogical usability	4.34 (0.77)		3.43-5.00	
Sociocultural usability	4.13 (0.87)		3.00-5.00	

**Figure 6.**
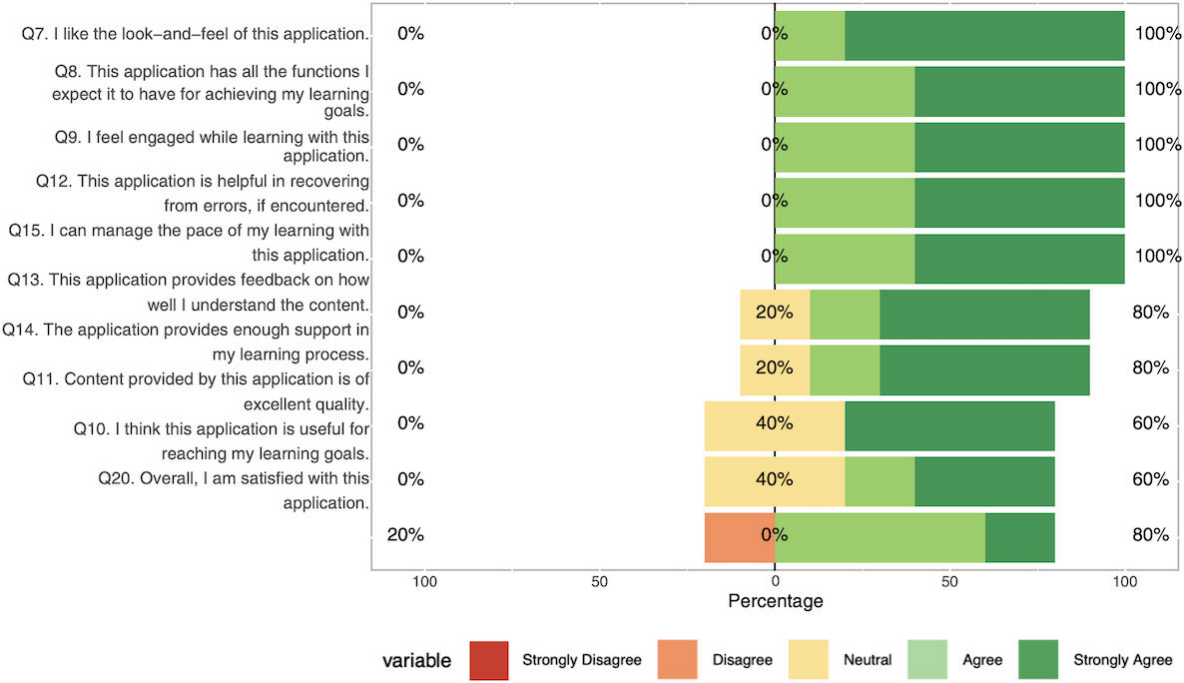
Pedagogical usability responses collected during usability testing using the Comprehensive Assessment of Usability for Learning Technologies (CAUSLT), showing domain and item-level means and SDs for pedagogical measures (ease of learning, clarity, learning support, engagement).

**Figure 7.**
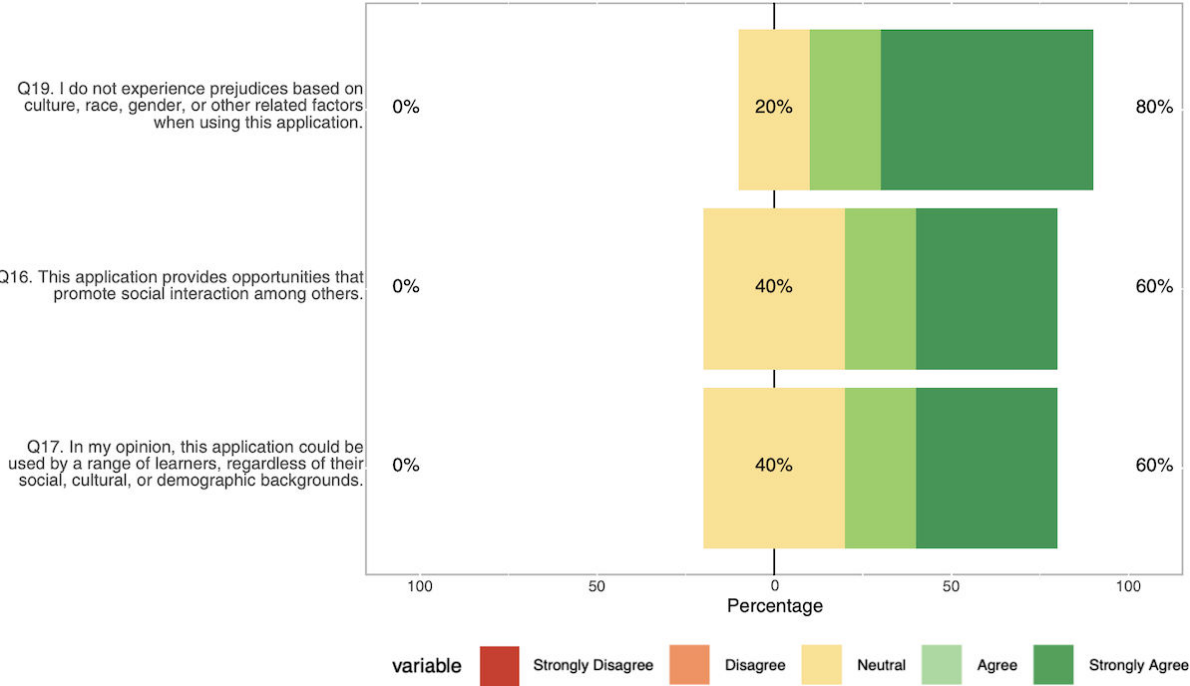
Sociocultural usability responses collected during usability testing using the Comprehensive Assessment of Usability for Learning Technologies (CAUSLT), showing domain means and SDs for sociocultural presence, accessibility, and relevance.

Pedagogical usability received the highest ratings (mean 4.34), with participants particularly valuing the application’s look and feel (mean 4.8) and core learning functions including engagement, error recovery, and pace management (mean 4.6). Of the participants, 100% (5/5) agreed or strongly agreed on items related to learning engagement, pacing, and functional adequacy, with only feedback-related items showing some neutral responses (1/5, 20%). Technological usability scored well overall (mean 4.06), with strongest ratings for ease of use, task completion speed, and accessibility across different abilities. We found 80% (4/5) agreement across most technological items, though navigation and user control received slightly lower ratings (3/5, 60% agreement), and content overwhelm was the only item receiving any disagreement (1/5, 20%). Sociocultural usability (mean 4.13) showed more variability. Participants were most confident about avoiding cultural prejudices (4/5, 80% agreement), while social interaction opportunities and cross-demographic accessibility both received 60% (3/5) agreement, with higher levels of neutrality (2/5, 40%), indicating potential areas for enhancement.

### Key Usability Findings

Qualitative analysis of think-aloud transcripts and observation notes revealed both significant strengths and areas requiring refinement in ePST’s usability. Content analysis identified patterns across participants’ experiences that highlight the application’s effectiveness at engaging users with TBI while revealing specific technical and interface challenges that impact user experience (Table 7). These findings provided actionable insights for iterative design improvements, which were incorporated between each usability testing session.

**Table 7.** Usability strengths and priority areas for improvement, summarizing positively rated features and recurrent problems identified from think-aloud protocols, observer notes, Comprehensive Assessment of Usability for Learning Technologies (CAUSLT) responses, and Community Advisory Board and Community Engagement Studio review.

Finding	Description
Usability strengths	
Intuitive interface design	Participants navigated the application easily and found interactive elements engaging. One participant described the storyline object as “pretty cool,” indicating positive reception of multimedia components.
Effective progress tracking	Badge system and progress indicators were clearly understood and valued by users. Representative quotes: “It looks like I’ve received one badge 1,2,3, and five more to go” and “It proves to me that I’ve done something.”
Engaging multimedia elements	Varied voiceover tones, storytelling approach, and visual design elements received positive feedback. Participants appreciated the narrative-based learning style and accessibility features.
Successful error recovery	Adaptive feedback mechanisms enabled users to recover from errors in knowledge checks without significant frustration, maintaining learning continuity.
Areas for improvement	
Mobile navigation issues	Users experienced confusion with mobile interface controls and activity progression. Representative quotes: “I see a button on the bottom right that looks like a back arrow” and “Wait, where was 3.2?”
Content comprehension	Some participants misunderstood instructions or content elements. One participant stated “Wait, this is not a question” when encountering a Storyline component.
Technical performance	Loading delays and playback issues disrupted user experience. Representative quotes: “Oh, it has to load all over again” and “I can’t see the whole screen” (mobile display problems).

## Discussion

### Principal Findings

We applied a CBPR and learning experience design–guided formative design process to develop and evaluate ePST and addressed 3 core questions about front-end needs, sociotechnical-pedagogical usability, and how identified issues were resolved through design refinements. Usability was high across all domains, knowledge accuracy was 80% (an encouraging result for formative testing suggesting acceptable immediate comprehension), and mean time-on-task was 11.47 minutes per lesson while engaging in the think-aloud protocol. Participatory activities produced concrete design changes (ie, microlearning 5-12–minute lessons, badge refinements, voiceover adjustments), helped identify partner-specific priorities (ie, caregiver, clinician, lived experience perspectives), and revealed TBI-specific requirements (ie, linear progression, higher technical performance, explicit content signaling).

Taken together, these findings suggest that sustained community engagement can yield measurable usability improvements and actionable implementation guidance for TBI mHealth interventions. These outcomes map directly to established digital health usability constructs of effectiveness, efficiency, and satisfaction (ISO 9241‐11) [121] and to mHealth-specific evaluation guidance such as the validated mHealth App Usability Questionnaire [122]. Our combined questionnaire plus think-aloud pipeline also follows human factors and usability engineering recommendations for medical and mHealth systems (IEC 62366) [123,124] and recent mHealth usability reviews [125,126].

### Iterative Community Feedback Enhanced Technical Usability

The Community Advisory Board structure (33 diverse community partners) and structured Community Engagement Studio sessions (n=10) enabled systematic integration of community input across development phases. CAUSLT scores averaged 87.3 out of 100, with pedagogical usability receiving the highest ratings. Participants completed lessons efficiently and achieved 80% accuracy on knowledge assessments, comparing favorably to cognitive rehabilitation intervention outcomes reported in systematic reviews [127]. Empathy interviews with TBI survivors revealed specific cognitive load concerns that directly informed the microlearning approach (5‐12–minute lessons) and progress tracking features. Community Advisory Board feedback on early prototypes resulted in modification of the badge system design and influenced voiceover tone selection to reduce perceived condescension. These modifications were fundamental design decisions that addressed cognitive accessibility requirements identified through community input [128]. Importantly, usability issues identified through think-aloud protocols mapped directly to areas where Community Advisory Board input had been limited or where technical constraints overrode community recommendations, suggesting that user involvement depth correlates with usability outcomes [129].

### Multistakeholder Representation Identified Comprehensive Design Requirements

The Community Advisory Board’s composition systematically identified design considerations that single partner approaches typically overlook. Caregivers identified family involvement features, while clinicians contributed evidence-based content validation, and individuals with lived experience prioritized autonomy and stigma reduction elements. This multiperspective input directly shaped the sociocultural usability features that scored highly in evaluation, particularly around cultural responsiveness and inclusive design [130]. Unlike traditional focus groups or surveys, the sustained Community Advisory Board engagement spanning the entire development cycle allowed for iterative refinement based on evolving understanding of user needs. This depth of engagement appeared to contribute to high pedagogical usability scores and enabled authentic relationship-building rather than extractive consultation [72].

### TBI-Specific Technology Design Requirements Emerged

The usability evaluation revealed specific design requirements for cognitive rehabilitation technology that extend beyond general accessibility guidelines. Analysis of user interactions demonstrated that traditional e-learning design principles require significant adaptation for users with cognitive impairments, consistent with cognitive load theory applications in special populations [131]. The 21-minute range in task completion times (range 4.6‐21.42 min) revealed that cognitive processing variability in TBI populations requires deliberate architectural choices rather than standard responsive design. Participants performed optimally with linear content progression and struggled with branching navigation structures, suggesting that linear content progression may reduce cognitive demands relative to complex navigation structures for users with executive function deficits [127].

Navigation issues identified in think-aloud protocols were predominantly mobile-specific, with participants reporting confusion about interface cues (“I see a button on the bottom right that looks like a back arrow”) and progression sequences. Technical performance issues disproportionately disrupted learning flow for participants with attention deficits, suggesting that cognitive rehabilitation technology requires higher technical performance standards than typical educational applications [132]. Although participants appreciated multimedia elements and voiceover variety, content comprehension issues arose when instructional clarity was sacrificed for engagement, suggesting that TBI rehabilitation technology might require explicit signaling of content types and interaction expectations, with clarity taking precedence over novel interface design [133].

### Methodological Contributions

This study contributes methodological insights for implementing CBPR in rehabilitation technology development. The integration of Community Advisory Board and Community Engagement Studio structures with learning experience design principles demonstrates how participatory research can move beyond consultation to systematic co-design. The sustained engagement model (33 diverse partners across the entire development cycle) provides a replicable framework for authentic community involvement, providing an actionable alternative to extractive research practices. The mapping of community input to specific design modifications illustrates how participatory methods can produce measurable technical improvements, not merely ethical and accessibility compliance. Findings support the claim that CBPR’s value extends beyond moral imperatives to offer practical advantages in rehabilitation technology effectiveness.

The literature consistently supports that technology development through iterative user-centered design is associated with higher adherence and lower abandonment [134-139]. This more frequent and consistent engagement leads to clinical benefits [134,140]. Additionally, high usability facilitates scale-up and sustainability [135]. Despite this, development of digital health care technologies often fails to include patient, client, and clinician voices through early and ongoing user-engagement [134,141,142]. A recent scoping review [139] on reasons for abandonment of behavioral and mental health mobile interventions found 6 categories of reasons for abandonment, 3 of which could be directly addressed through user-centered and participatory design: (1) poor user experience, (2) evolving user needs and goals, and (3) content and features.

There is a growing body of literature specifically in rehabilitation supporting that usability, acceptability, and user-centered design contribute to implementation and sustainability of remote, technology-support interventions [28,143-146], but substantial work still needs to be done. A systematic review of cognitive rehabilitation interventions for older adults found that usability and user experience often explained mixed effectiveness of these technology-based interventions [147]. Though an even smaller body of research, a few studies have examined user-centered design for assistive technology and cognitive rehabilitation interventions for people with TBI [28,143-146]. These papers, consistent with our own findings, emphasized the importance of (1) tailoring the technology to reduce cognitive load; (2) having high error tolerance and easy error correction; (3) including multimodal prompts; and (4) involving clinicians, care partners, and survivors in technology design. Evidence in TBI is smaller and more heterogeneous than in general digital mental health, but findings consistently point to usability as a facilitating factor for adoption and benefit.

This study’s contribution is integrative rather than disciplinary. We operationalized a full-cycle pipeline that combines community-based participatory research with learning experience design; mapped participatory inputs onto a sociotechnical-pedagogical evaluation lens; and triangulated think-aloud, task, and survey metrics to produce community-informed design principles for TBI mHealth. Taken together, this cross-disciplinary operationalization provides a reproducible, pragmatic approach for formative mHealth development in cognitive rehabilitation and offers concrete, testable design guidance for teams working at the intersection of participatory methods, instructional design, and digital health.

### Limitations and Future Directions

Several limitations constrain the generalizability of our findings. Usability testing used a small, purposive sample (n=5) appropriate for formative evaluation but insufficient for population-level inferences. Consequently, the effect estimates (eg, CAUSLT mean, accuracy) had wide confidence intervals; therefore, subgroup effects could not be assessed. Thus CAUSLT mean, knowledge accuracy, and completion rates should be viewed as exploratory. Our design mitigations were purposive sampling for heterogeneity, triangulation across qualitative and quantitative data streams, and sustained Community Advisory Board engagement to improve ecological validity. Nonetheless, future work should evaluate ePST in larger, more diverse TBI samples to quantify variability across injury characteristics, device types, demographic groups, and contexts of use and to permit powered hypothesis testing and subgroup analysis, which is the focus of our current feasibility study. Further, some reported technical issues may reflect device-specific limitations rather than design flaws, indicating need for expanded cross-platform testing. In addition to this, the TBI-specific design features reported here may not transfer directly to other neurological populations, requiring investigation of how CBPR-based approaches perform across different rehabilitation contexts. Although initial usability testing revealed strong satisfaction, sustainability of engagement remains unknown, suggesting a need for longitudinal metrics capturing retention, adherence, and health outcome durability. Future research should focus on evaluating barriers and facilitators to adoption and abandonment and how this engagement (or lack thereof) affects scale-up and sustainability of health care technologies using digital health frameworks such as the NASSS (nonadoption, abandonment, scale-up, spread, and sustainability) framework [135].

A direction for future research is how participatory practices might influence long-term health outcomes and treatment adherence beyond usability metrics. Integration of adaptive technologies such as artificial intelligence–driven personalization, voice-guided prompts, and real-time support could represent promising directions for accommodating cognitive variability in neurological populations. Additionally, examining the scalability of intensive CBPR approaches across diverse rehabilitation contexts (ie, stroke recovery, spinal cord injury) could advance understanding of participatory design’s broader applicability.

### Conclusions

This study demonstrated that systematic application of CBPR principles can produce both qualitative and quantitative improvements in rehabilitation technology usability through iterative community feedback, diverse stakeholder representation, and sustained engagement processes. The development of ePST illustrates how participatory methods can address specific design requirements for cognitive accessibility while maintaining high user satisfaction. The findings suggest that cognitive rehabilitation technology can benefit from specific design considerations including attention to cognitive load, clear navigation patterns, and explicit content signaling to address TBI-related challenges. This work provides further support for CBPR as a practical methodology in rehabilitation technology development, enhancing ethical research practices as well as technical outcomes. Investigation of long-term engagement sustainability and adaptive technology integration remains a direction for future research with promise for advancing understanding of how participatory approaches might contribute to more equitable, personalized, and effective rehabilitation interventions.

## Supplementary material

10.2196/83995 Multimedia Appendix 1 Traumatic brain injury (TBI) personas and empathy maps for user-centered design: 11 de-identified, data-informed personas and associated empathy-map summaries created from Community Advisory Board (CAB)/Community Engagement Studios (CES) and empathy-interview data to guide Electronic Problem-Solving Training (ePST) module design and accessibility decisions. Each persona includes demographics (age, language, location), TBI characteristics and time-since-injury, goals, behaviors, attitudes, motivations, barriers, facilitators, and concise “key attributes” used to prioritize features (eg, linear lesson flow, memory supports, fatigue accommodations, family involvement).

10.2196/83995 Checklist 1 GRIPP 2 Short Form checklist.

## Abbreviations

CaDeS: Caregivers in Dementia PST and DSJ
CBPR: community-based participatory research
ePST: Electronic Problem-Solving Training
mHealth: mobile health
NASSS: nonadoption, abandonment, scale-up, spread, and sustainability
PST: Problem-Solving Training
TBI: traumatic brain injury
