# Supplementary material for: Designing Electronic Problem-Solving Training for Individuals With Traumatic Brain Injury: Mixed Methods, Community-Based, Participatory Research Case Study
Source: J Med Internet Res. 2026 Jan 20;28:e83995. doi: 10.2196/83995 (PMC12818508; doi:10.2196/83995)
Supplement: Multimedia Appendix 1 [file jmir-v28-e83995-s001.pdf]

TBI Personas and Empathy Maps for User-Centered Design

Matthew Schmidt, PhD[1]
Yueqi Weng[1], BS
Shannon Juengst[2], PhD, CRC
ePST Community Advisory Board*

[1]University of Georgia, Athens, GA
[2]Brain Injury Research Center, TIRR Memorial Hermann, Houston, TX
University of Texas Health Science Center at Houston, Houston, TX

ePST Community Advisory Board Partners

Carly Almy, PhD, OTR
Chen-Yi Cheng, BS
Jacob Finn, PhD
Christina Gullickson, BA
Alexandra Holland, LMSW
Novelle Kew, PhD
Kate Marshal, MA
Monique Pappadis, PhD, MEd
Nicholas Pastorek, PhD, ABPP
Jonathon Richter, EdD
Angelle Sander, PhD
Sara Freeman Smith, MA
Sierra Tobia, BSN
Michael Williams, PhD
Jay Bogaards, MA, CCRP
Haleigh Cushen, MEd, CRC
Luis Gonzales, BS
Isabel Gullickson
Ryan Holliday, PhD
Adam Kinney, PhD, OTR/L
Candice Osborne, PhD, MPH, OTR
Kristin Parks, MEd
Peggy Reisher, MSW
Gabriel Rodriguez, PhD
Sara Shugars, MS, CRC, CCM
Stephen Sutter, BS
Shari Wade, PhD
Kristin Wilmoth, PhD

Alexis

Balanced Contemplator

"Your injury does not define you."

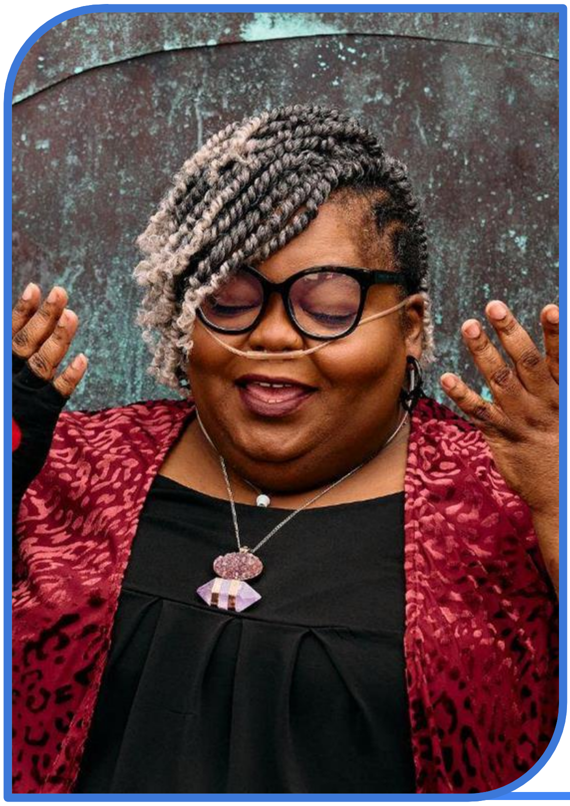

**Time Since Injury**	6 years
**LANGUAGE**	English
**AGE**	59
**JOB**	Unemployed
**INCOME**	$0
**RACE**	African-American
**EDUCATION**	GED
**STATUS**	Single, Divorced
**LIVING WITH**	Alone with regular support from children
**LOCATION**	Rochester, MN
**TBI CHARACTERISTICS**	balance issues, an unsteady gait, muscle weakness, and coordination problems, necessitating the use of assistive devices and home modifications to maintain her independence and safety

GOALS

- Being respected and understood by family and others
- Having others expect her to be self-sufficient
- Be capable of doing basic things in daily life independently

BEHAVIOR

- Spends 3 hrs/day exercising for physical recovery; has muscle weakness and balance issues
- Learned basic things again (e.g., walking, speaking, eating)
- Moderating her emotions when she feels stress
- Using note-taking and phone recording for reminders
- Doing things consistently (e.g., placing things in a certain order or specific place)

ATTITUDE

- Wants to be independent and for others to have expectations of her
- Believes recovery is a gradual process
- Feels people with similar experiences can understand each other better
- Trust that therapies can help her to recover

MOTIVATION

- Wants to improve outcomes through a positive mindset.
- Wants to strengthen memory through tools, strategies, and family/friends' support.
- Empathize with people through their stories

KEY PERSONALITY ATTRIBUTES

- Persevering
- Very dependent on others
- Wants to be understood
- Receptive to help from others

BARRIERS

- Takes a long time to learn something
- Understanding directions for taking medications
- Gets confused when things are not where they go

FACILITATORS

- Technology
- Family/friends' support
- Visual reminders
- Hearing others' stories

Alexis Reed

Say

- Mine was. I had to learn how to walk, eat, brush my teeth, and move and everything, even talk all over again.
- I had forgotten how to do everything.
- [Recovery] was quite difficult
- My family members, they weren't understanding how I could be grown and not remember how to do the basic things.
- I'm like a baby. I have to learn all over again, and then me getting frustrated.
- Every morning is like starting from zero, trying to piece together who I am.
- The physical therapy sessions are exhausting, but I keep being told they're crucial for my recovery.
- Sometimes, I just sit and try to remember my life before the accident, but it's like looking through fog.
- I've had to use sticky notes around the house to remind me of simple tasks, which is both helpful and disheartening.
- Talking to others who have gone through similar experiences has been a lifeline for me.

Think

- Reflects on her journey and progress as a process
- Expresses concerns and hopes about her future
- Contemplates her identity and changes in her life post-injury
- Shares feelings about support systems (or the lack thereof)
- Ponders her interactions with and understanding (or misunderstanding) from others
- Finds comfort and understanding through others' stories
- Develops practical approaches to managing daily life (i.e., strategies for remembering everyday tasks)
- Acknowledges emotional responses to recovery setbacks and challenges
- Understands that she must accept and adapt to changes post-injury

Do

- Engaged in intensive therapy to relearn basic motor skills and daily routines.
- Started from the beginning to reacquire fundamental life skills.
- Faced new challenges every day, adapting strategies to cope with and overcome them.
- Attempted to explain her situation to her family, seeking empathy and support.
- Repeatedly practiced everyday tasks, dealing with the frustration of slow progress.
- Worked on rebuilding her identity, reflecting on her progress and setbacks each day.
- Persisted through exhausting physical therapy sessions, focusing on long-term recovery goals.
- Spent time in reflection, attempting to connect with memories from her life before the injury.
- Implemented practical strategies like using sticky notes to manage daily tasks and reminders.
- Sought support and shared experiences with others in recovery, finding comfort and inspiration in their stories of resilience.

Feel

- Overwhelmed by the daunting task of relearning basic functions, yet determined to recover.
- Disoriented by her loss of memory, grappling with the reality of starting anew.
- Frustrated with the unexpected difficulties, but resilient in facing them.
- Hurt and isolated by her family's lack of understanding, yearning for empathy.
- Embarrassed and impatient with herself, struggling to accept her new reality.
- Anxious about her identity and future, but hopeful as she makes small strides.
- Physically drained yet mentally steadfast in the belief that recovery is possible.
- Nostalgic and sorrowful for her lost memories, clinging to hope for clarity.
- Grateful for the aids that help her manage, yet disheartened by her dependence on them.
- Comforted and inspired by the shared experiences, feeling less alone in her journey.

Pains

- Struggling with the basic tasks of daily living that were once second nature, leading to feelings of frustration and disorientation.
- Experiencing a lack of understanding and empathy from family members, which adds to the sense of isolation and hurt.
- Facing the daunting reality of starting from scratch, with every morning feeling like starting from zero in rebuilding her identity and capabilities.
- Dealing with physical exhaustion from intensive therapy sessions, which are necessary but taxing on her stamina and morale.
- Grappling with the loss of her past self and memories, leading to moments of nostalgia and sorrow for what was lost in the accident.

Gains

- Gradually reacquiring fundamental life skills through therapy, demonstrating resilience and determination in the face of adversity.
- Building a new sense of identity and understanding of herself, reflecting on progress and setbacks with a hopeful outlook for the future.
- Implementing practical strategies, like using sticky notes for reminders, which help manage daily tasks and contribute to regaining independence.
- Finding comfort and inspiration in connecting with others who have gone through similar experiences, reducing feelings of loneliness.
- Holding onto hope and the belief in recovery, fueled by small strides and the support of the recovery community, showing a path forward amidst challenges.

Jordan

Resilient Maverick

"Quote and unquote disabilities are my strengths."

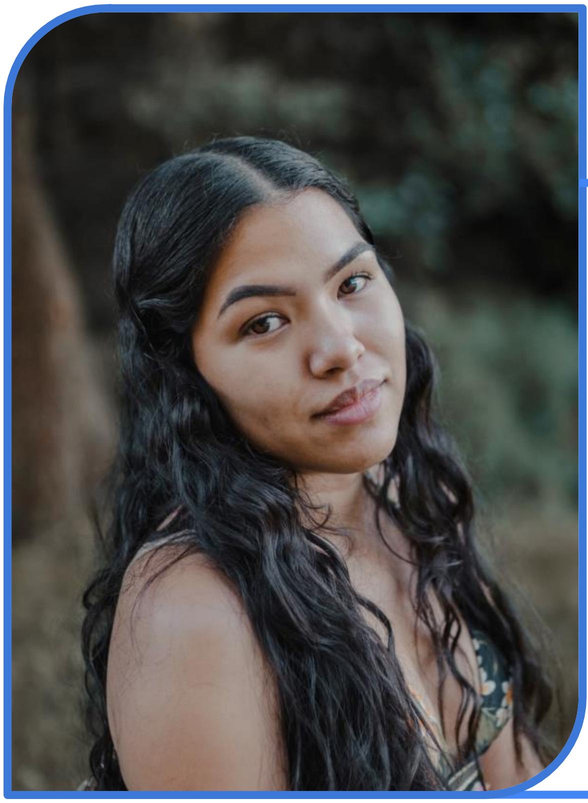

**Time Since Injury**	12 years
**LANGUAGE**	Spanish, English
**AGE**	32
**JOB**	Teacher, health interpreter
**INCOME**	$80,000
**RACE**	Latina
**EDUCATION**	Undergraduate
**STATUS**	Married
**LIVING WITH**	14 year-old daughter, 40 year-old husband
**LOCATION**	El Paso, TX
**TBI CHARACTERISTICS**	memory issues, chronic fatigue, and concentration difficulties, needs to use reminder systems and adaptive strategies, music and dance for stress, prefers self-reliance over medical treatments

GOALS

- Be able to achieve the level of function she had before having a TBI
- Be perceived as someone who is not disabled
- Be effective at her job

BEHAVIOR

- Alternates her work with rest because she frequently struggles with fatigue and concentration issues, making it challenging to maintain her workload.
- Learned and uses approaches such as repetition and associating with something during her work to strengthen her memory.
- Relieves stress and negative emotions (e.g., anxiety) through music and dance
- Visited doctors at first, but then overcame the barriers by herself

ATTITUDE

- Values therapies that can help her recover, but is skeptical about medical therapy
- Feels others' TBI experiences are not relevant because her experience was so unique
- People with disabilities should not be labeled
- Struggles with accepting help from others because she wants to be self-sufficient, often feeling isolated because of her unique experience

MOTIVATION

- Wants to improve outcomes through a positive mindset.
- Wants to strengthen memory through tools and strategies.
- Determined to prove that she can succeed professionally despite her TBI.
- Drivesn to find innovative solutions to cognitive challenges

KEY PERSONALITY ATTRIBUTES

- Self-sufficient
- Excellent communicator
- Unflappable
- Independent thinker

BARRIERS

- Reluctant to socialize because it is hard to remember names and she gets tired easily.
- People with disability or with special needs are discriminated against or labeled.
- Not positive about medical therapy for TBI
- Struggles with fatigue and concentration, which affects her productivity and job performance

FACILITATORS

- Belief that people with disabilities shouldn't be treated differently
- Music and dance
- Job and colleagues

Jordan Lee

Say

- So I will had to be full force cognitive, and then some and I just found the key using the monic devices even something as simple as like grade level.
- so I'm horrible in names, but now I do also learn to associate with something. So that'll help me, remember.
- I see myself like I can do it just I actually have to work at it now.
- Or even if I'm in the public place, I can. But in my head. I'm I'm dancing, and I'm just doing this. ... like peaceful time like God help me out! Let me help me deal with this things that nature.
- At the beginning I was going to psychologist, psychiatrist, and I was on antidepressants at the beginning. But now my my nurtures have override that, and I'm no longer on any medicines.
- Yes, overall writing it down does help because it's come to visual. And sometimes you're seeing it, says Audio, more ways of learning it.
- I don't relate to people cause you made a walk and choose like mine, but they weren't exactly my shoes. So I know we're very different.
- I think it'd be easier if it's not label for someone. Special needs kind of thing. It's just for all.
- My quote, unquote disabilities have given me strength.
- I had to take my driver license exam again at age 19, due to liability.

Think

- Believes that she can use tools to support her memory challenges (i.e., mnemonic devices, note-taking)
- Believes that she is fully capable of doing what she puts her mind to, but understands that it may require more effort than before her injury
- Prefers to say that something is designed for "all", not specifically for those with disabilities.
- Believes that people with disabilities or special needs should not be labeled.
- Admitted the disability change her life, but it is also an advantage and an asset.
- Believes that learning about other people's experiences with TBI is not very useful because her experience was so personal and unique.

Do

- Used mnemonic devices to help her study and work.
- Learned and used approaches such as repetition, and associating with something during her work to strengthen her memory.
- Relieved stress and negative emotion (i.e., anxiety) through music and dance
- Reminded herself by note-taking (writing things down)
- Careful about language use to avoid offending people with disability.
- Accepted the fact that the disability brought changes to her life and used it in a positive way.

Feel

- Does not like terms like special needs or disabilities.
- Does not feel entirely positive about medical help (psychiatrist, psychologist) or medicine, but sees value in cognitive behavioral therapy
- Uncomfortable with how neurotypical people relate to people with disabilities/special needs, i.e., treating them differently by default.
- Open to talk about the pros and cons that disability brings to her life.

Pains

- Hard to remember names
- People with disability or with special needs are discriminated against or labeled.
- Believes that she has recovered, but others still see her as having a disability.

Gains

- Learn to use tools to strengthen the memory
- Learned to use strategies like repetition and association to help her with her work responsibilities as a health interpreter
- The "quote and unquote disabilities" are seen as an advantage and an asset

Taylor

Resourceful Innovator

"Turning adversity into opportunity."

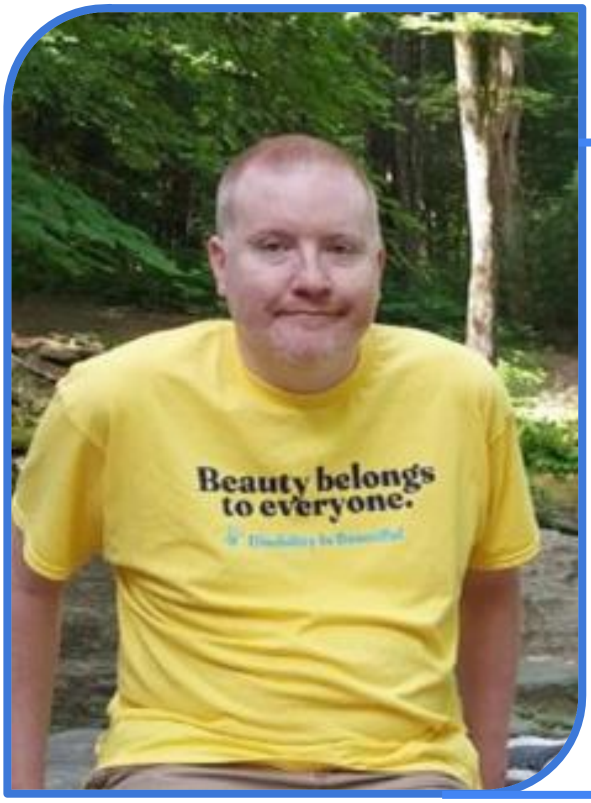

**Time Since Injury**:	5 years
**Language**:	English
**Age**:	42
**Job**:	Veteran, seeking employment, previously in IT
**Income**:	Exploring opportunities for self-sufficiency
**Race**:	White
**Education**:	Bachelor's Degree in Computer Science
**Status**:	Single, but closely connected to family
**Living With**:	Alone, with support from family and friends
**Location**:	Little Rock, AR
**TBI Characterization**:	Severe TBI involving a period of coma and extensive rehabilitation, resulting in significant memory and cognitive challenges that require ongoing adaptation and use of technology for daily tasks and organization.

GOALS

- Achieve professional reintegration and financial independence.
- Foster strong relationships within the TBI community.
- Advocate for awareness & adaptive technologies in TBI recovery.

BEHAVIOR

- Participating in local and online forums, speaking at events, and collaborating with organizations to raise awareness about TBI.
- Adopts technology as a natural extension of his daily life to manage tasks and stay organized.
- Engages in regular physical activity for mental and physical health benefits.
- Actively participates in peer support groups, offering support and gaining insights from shared experiences

ATTITUDE

- Embraces technology and innovative strategies for memory and organization.
- Views his TBI recovery journey as a platform for growth and resilience, but accepts shortcomings.
- Strong belief in the power of community and peer support for mutual recovery benefits

MOTIVATION

- To regain independence and professional fulfillment.
- To continuously improve memory through adaptive strategies.
- To inspire and assist others with TBI by sharing his journey and strategies.

KEY PERSONALITY ATTRIBUTES

- Continue to reality of "new normal"
- Innovative
- Open-minded
- Goal-oriented

BARRIERS

- Memory and cognitive challenges requiring ongoing adaptation.
- Continues to navigate societal perceptions and self-identity post-TBI, even after reaching self-acceptance.
- Balancing independence with the need for support.
- Hard to recall and think due to the coma in his previous work

FACILITATORS

- Technology and adaptive tools for reminders and problem solving.
- Personal drive and positive outlook towards recovery and adaptation.
- Daily interactions with a supportive community that provides emotional and practical assistance.

Taylor Cameron

Say

- Yeah, I think that my, the what I use most that helps me the most is voice to text. So it helps me say it out loud and then read it. and then email it, or text it to myself, and then add it to my calendar. So I get the repetition.
- I'm awful with names. And so whenever I was at tier. What I do is I focus on one feature or attribute about the person that I recognize. That's different than other things that I don't recognize and other people, and then I just repeat their name, and then that helps me.
- getting upset or emotional side effects. My after my head. Injury is primarily memory. I just couldn't. I couldn't remember anything. So I don't really have any. Input on the methods are things that had not helped me with emotional effects.
- writing things down is key for me.
- repetition and writing it down for some reason helps it stick, and I use the notepad on my iphone to do it sometimes, but if I have the piece of paper I carry a little.
- So for me listening to other people's stories that I've never there's never been anything bad to come from. That is always been awesome. I loved it.
- can't do something that I think I should be able to do
- when you share your like, people think it's awesome, so like and overcoming what we've all been through. I don't see it as like a negative thing. I think it's a blessing. It's a miracle

Think

- Do not have methods that can help him with emotional effects
- Phone and notebook are helpful for him to relate and remember things
- Relying on Vitamins and some supplements as placebo
- Can imitate the successful people by setting the goal for each day
- People need and want to have the outlet so that they can talk to
- Had family to lean on or others' support help him most
- Hearing from others' stories help him when he needs support or encounter the similar things.
- Everyone has their preference to use different tools to support them remember things. Some people keeps the conspiracy theories in mind and fear of using technology will impede their abilities to normal life.
- Acknowledged that everyone' TBI is different. Some people may not find something helpful from others' stories.
- Want the learning program can help to strengthen his memory and able to control his temper himself.

Do

- Use technology such as voice-to-text, email, calendar to help him remember things
- Focus on one feature or attribute of people to recognize them
- Repetition and writing things down help stick his memory (using phone and paper)
- Take Vitamins and other supplements as the placebo
- Doing exercise to let him sleep better
- Found the spiritual nourishment through reading the book. Learning the successful people to set the goal for each day
- Writing down the goals so that they can remind him
- Noticing he can't do something he believes he can do, he will try his best to do things right.
- Don't see himself as what he hear the negative things from others

Feel

- Felt awful with names
- Felt helpful when taking Vitamins and some supplements because of Joe Rogan's saying "Alpha brain makes your memory work that better".
- Excited to share his approaches to strengthen his memory. (i.e. exercise, reading books and learning from successful people)
- Like hearing from others' stories which can help him, and he can learn from others' experience.
- Glad to be recognized and praised by others when sharing his stories.
- Felt degraded but behaved positive when someone said negative things about him.
- Realizing both sides of the improvement of TBI experience because the state of Arkansas has started to extended the medicare.

Pains

- Doing bad at recognizing people and remembering things
- Can't do something that he thinks he should be able to do
- Got his mind messed up after the accident
- Don't have insurance, don't have a job.
- Being treated differently. (hard to get the driver license; being said negative things)

Gains

- Use his approaches to remember things (i.e. using technology and handwriting to remember things; using features or attributes to recognize people)
- Find the spiritual treatment that motivate him to stick something in mind
- Learning and gaining supports from hearing from others' stories

Casey

Adaptive Veteran

"Embracing tradition in a digital world."

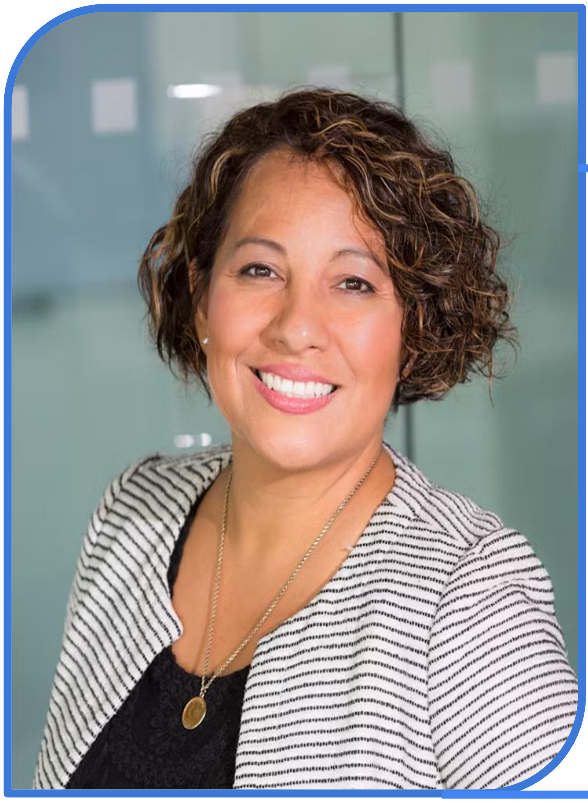

Time Since Injury: 25 years
Language: English, Spanish
Age: 44
Job: Worked in TBI support since 2016
Income: Not specified
Race: Latina
Education: Bachelor's and Master's degrees in Social Work
Status: Single, but closely connected to family. Served in the military for 5 years before injury
Living With: Son, but in a separate building
Location: Miami, FL
TBI CHARACTERISTICS: Memory impairment, attention deficit, executive dysfunction, mood swings, stress-related headaches, sensory overload (prefers quieter settings). Manages symptoms with planners, strict routine, humor, pragmatism.

GOALS

- To share her experiences and strategies with the TBI community.
- To encourage a more understanding and inclusive approach to disability.
- To advocate for Veteran issues and provide support for fellow Veterans with TBI.

BEHAVIOR

- Uses traditional planners and calendars for daily tasks and reminders, not smartphone apps.
- Open about her memory challenges, adopting a straightforward and humorous attitude towards them.
- Actively engages in learning and teaching in social work, leveraging her experiences to educate others.
- Participates in Veteran support groups and attends Veteran events.

ATTITUDE

- Prefers traditional methods of organization & learning, like writing things down in a planner, over digital tools.
- Approaches challenges with humor and pragmatism.
- Believes in the mutual benefit of sharing with and learning from others.
- Has a strong sense of duty and camaraderie towards fellow veterans.

MOTIVATION

- Driven by a desire to continue functioning effectively despite TBI.
- Seeks to empower others with TBI through her work and personal example.
- Aims to bridge the gap between traditional and digital methodologies.
- Committed to improving the lives of Veterans, particularly with TBI.

KEY PERSONALITY ATTRIBUTES

- Resilient
- Practical
- Humorous
- Knowledgeable
- Dedicated

BARRIERS

- Taking time to concentrate and learn to use technology to know the international news.
- Overcoming the stigma and challenges associated with memory impairment.
- Balancing the desire to contribute with the need for observation and reflection.
- Dealing with the unique challenges faced by Veterans, such as PTSD and accessing Veteran-specific resources.

FACILITATORS

- Strong academic background providing a foundation for understanding and working with TBI.
- Community and professional environments that value her experience and perspective.
- Personal adaptability and the willingness to learn from others.
- Support from Veteran organizations.

Casey Jordan

**Say**<br>• And honestly, I prefer to still write things down in like a calendar or a planner or stuff like that<br>• I would much better rather write it down, because then you get to see it as you're writing it, and you have to reread it to make sure you wrote it down right, and typing it in is just useless to me, especially on my iphone, because it will automatically complete the words for you. And so you don't even really have to think<br>• Well, my experience I guess, might be a little bit different, because I got my bachelors and my master's degree in social work<br>• So I went to school to learn how to work with me<br>• and so all the other students thought I was awesome, and they wanted to hear all about it, cause they were learning from me. and I was learning from them. So it was great.<br>• I should have gone into social work.<br>• The most important thing that I learned was that yes, I can still do it. It might. I might do it a different way, but I can still do it	**Think**<br>• Believed that writing things down are better to strengthen and support her memory because she needs to confirm and reread what she wrote down were correct<br>• Kept the belief that the phone to her is to make the phone calls<br>• Should go into social work earlier since Doing the social work can help her learn from the others<br>• Though being affected by TBI, she will do differently in her life than only accepting the fate.
**Do**<br>• Learned to write things down to help remember the things<br>• Use phone to only make the phone calls<br>• Went to school to learn how to work with the people who are similar to her<br>• Learned from her cohort and students during the social work<br>• Do not remember how she dealt with her memory during the 25-years life and recovery experience	**Feel**<br>• Felt using the technology is another learning process.<br>• Felt encouraging by being praised by her students that she was awesome. And others also learned from her experience<br>• Determined and fortunate that she still insists in the social work<br>• Felt fortunate and encouraging that she can still do something different despite of TBI experience

Pains	Gains
• The accident happened early. The traditional way of taking note is the only way to help her to strengthen the memory<br>• Her memory is a barrier in her work and life	• Find her motivation in social work. The motivation drove her to make changes from her TBI experience by going to school and working in more social-oriented field.<br>• Felt fortunate and grateful that she still can make changes throughout her life regarding the TBI experience

Riley

Accommodating Empath

"Thinking more before acting."

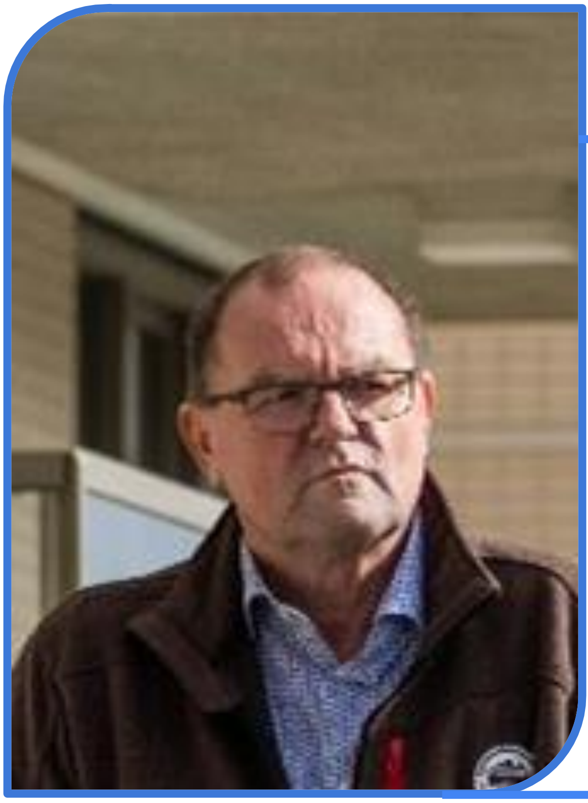

Time Since Injury: 15 years
Language: English
Age: 64
Job: Carpenter
Income: $47,000/yr
Race: White
Education: High school
Status: Single, divorced
Living With: Lives with his daughter
Location: Lancaster, PA
TBI Characterization: Severe TBI with long-term impacts on memory and emotional regulation, requiring the use of technology like phones and notepads for reminders and strategies to manage emotions effectively.

GOALS

- To be able to regulate his emotions
- To not be treated or labelled as someone with special needs
- To use his experiences to help people with similar experiences

BEHAVIOR

- Uses phone and notepad for reminders
- Regulates his emotions by walking away and calming himself down.
- Puts things into perspective by considering "what if others' experiences happened to me?"
- Treats people (i.e. his daughter) with special needs the same as anyone else
- Setting the short-terms goals for his life and problem-solving.

ATTITUDE

- Avoids expressing negative emotions so he won't impact others
- Believes spiritual wisdom can help with controlling emotions
- Believes that others' experiences can help him empathize
- Tries to stay positive and use humor

MOTIVATION

- Hearing others' stories helps him to frame things differently
- Being treated differently by others has led him to try to treat everyone the same

KEY PERSONALITY ATTRIBUTES

- Open to learning new strategies
- Accommodating
- Empathetic
- Considerate

BARRIERS

- Struggles with memory more and more as time goes by
- Being treated differently due to his disability
- Hard to participant in social activities due to the exhaustion and slow cognitive processing and reaction

FACILITATORS

- Technology such as phone and notepad
- Sharing stories to help others
- Open-minded personality

Riley Brooks

**Say**<br>• But I use my phone a lot, for I have not pad that I use my alarms because I can't remember anything as far as time goes<br>• But I use the notepad a lot, too, on just trying to help me remember something that II need to know later<br>• but a lot of times when my frustration level would got got so mad and I was around other people, I would just walk off and and go calm down myself, because I knew I needed to get away<br>• but hearing the stories from other people on on their experiences helps me think about looking at things in, in, in. In a better way<br>• When I was in high school I was in special needs classes, so everybody treated me differently because I was in special needs classes<br>• I learned from that myself. So in a way, they helped me instead of downing me, they actually kind of really helped me look at things the way I did, because my step, daughter, I'm divorced now.<br>• I'm Tbi forgot no what you call it. Recently I did a podcast as a many, many organizations. But it was focus on Latinos. No cause, really. They're saying exactly what I want to say.<br>• Actually, my, with, with my disability. I've actually learned more how to deal with things in life now than I was before my brain injury. Now, now cause now I think about things more. before I react.	**Think**<br>• Use technologies such as phone and notepad can help him remember the things he will do later<br>• Being spiritual wise can help to relieve the negative emotion<br>• Hearing from others' stories help him look things in a better way. For example, others' experience make him think about what's wrong or what's going on with him<br>• Recalled his experience in high school. Everyone treated him differently because he was in the special need class<br>• Should not label the people with special needs. (After being treated differently and hearing from other participants' stories.)<br>• Learned more about how to deal with things in life before TBI.<br>• Doing as much they can to help the next person with similar experience out.
**Do**<br>• Uses phone and notepad a lot to help him remember things.<br>• Walked off from other people and calmed himself down when he got frustration.<br>• Treat his daughter like father to daughter instead of the people with special needs.<br>• Share his TBI experience in a humorous way (like a funny story).<br>• Did a podcast focusing on Latinos with no reason. The Latinos said what he wanted to say.<br>• Taking into others' speakings (the ones with similar experience to him) and keep this belief	**Feel**<br>• Felt emphasized when hearing others' stories. For example, hearing from others' experience make him reflect what's going on with him.<br>• Reflected his experience in special need class when he was in high school in a positive way. (i.e. people helped him instead of downing him)<br>• Willing to share his experience on TBI to help the people with similar experience.

Pains	Gains
• Cannot remember things as time goes by<br>• Being treated differently as the people with special needs in high school	• Hearing from others' story can him to contemplate things in a different way. For example, he will think what he will do if others' experience happen on him<br>• Know how to deal with the people with special needs. For example, his daughter.<br>• Learn more skills in dealing with things in life before TBI. Thinking more before acting<br>• Switching the position that being treated differently by others is they do not want to let him down

Morgan

Positive Stabilizer

"Working hard to change life and be happy."

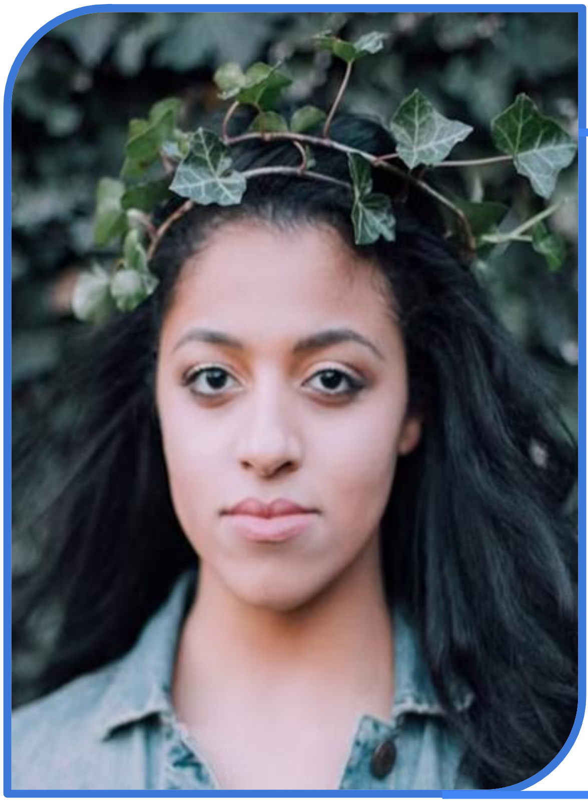

Time Since Injury: 11 years
Language: English, Spanish
Age: 29
Job: Part-time Comcast technician
Income: 13 dollars/ hr
Race: Latina
Education: Some Community college (GED)
Status: Married
Living With: Wife
Location: El Paso, TX
TBI Characteristics: Memory and behavioral regulation challenges; Easy to feel tired, Stutter and aphasia sometimes; cognitive fatigue, sensory sensitivity, and occasional anxiety, requiring the use of meditation for managing daily tasks and emotional stability, along with support from her wife.

GOALS

- To finish her associate's degree
- To be able to control her behaviors and emotions
- To not be treated differently just because she has special needs

BEHAVIOR

- Did some college classes in Houston, but gave up because of the exhaustion, affective and cognitive challenges.
- Uses meditation to help regulate her behaviors and emotions
- Asks people not to treat her differently just because of her disability.
- Sought help from her wife to study for her college classes

ATTITUDE

- Wants to go to college. However, gave it up because it is so taxing and takes lots of time.
- Doesn't want to be rude or mad
- Prefers to use technology to help her remember things
- Gets insights from others' stories
- Doesn't want to be labeled
- Tries to stay positive

MOTIVATION

- Regrets not finishing at UT; wants to finish college classes.
- Doesn't want to be rude to others
- Wants to overcome barriers with help from others

KEY PERSONALITY ATTRIBUTES

- Worried that TBI sequelae will not improve any more
- Independent thinker
- High self-esteem
- Friendly

BARRIERS

- Difficulty remembering things and communicating, and spending more time learning constantly
- Difficulty regulating her behaviors
- People treat her differently because they think there is "something off" about her because of her stutter and aphasia
- Difficulty identifying solutions to the problem

FACILITATORS

- Taking notes, flash cards
- Her wife supports her with community college classes
- Positive attitude

Morgan Taylor

Say

- Since it happened when I was so young I hadn't gone to college yet.
- like when I just need to remember something, I'll take notes or I'll set alarm.
- I personally think typing it on my phone is the most effective or just typing it out.
- Ii was getting super frustrated, and it was making me pretty upset because I'm not a like a mean person. and I was reacting very rude to like, think that would happen.
- And so I started doing meditation. and I meditate pretty much multiple times a day. And then I also I also adapted saying it is what it is, you know.
- being labeled like special needs or like I struggle with that quite a lot. just because I feel like a lot of people like strangers can tell that there's something off about me.
- I'm just as smart as them like I just have delay in my learning abilities in my speaking abilities.
- it's not very positive, and so I try not to think it, and I try not to let it into my life.
- there's not nothing you can do. Go back and change it. It. It happened, you know, you just have to accept it and move on and work really hard to have a happy life, and then everything's fine.

Think

- Chose not to go college based on her learning experience and financial condition.
- Pondered other's answer and combined her therapy experience to talk about her thoughts on the impact of hearing others' stories.
- Believes that typing things is more effective for her because of her personal preference to use technology
- Believes the aim of sharing stories and hearing stories from others is to learn what works for them.
- Accepts her experiences and tries to be positive.

Do

- Went to the community college classes and did note-taking, flash cards, repetition, and parents' support to help study.
- Took notes and set alarms to cope with stuff in her daily life.
- Did meditation multiple times a day to regulate her emotions.
- Felt comforted from her wife's words when she felt as if she were being labeled.
- Got rid of negative thoughts and feelings and tries not to let them into her life.
- Accepted the fact that she has a TBI and continues life with a positive attitude.

Feel

- Felt frustrated, upset, and regretful when she was mean and rude to others
- Willing to learn by hearing the stories of how others' deal with TBI
- Struggled with being labeled as special needs or someone with a disability.
- Uncomfortable about when someone can tell that there is "something off" about her.

Pains

- Cannot go to college because of the accident.
- Hard to control negative emotions.
- Struggles with being labeled as special needs or having a disability.

Gains

- Able to regulate herself when being upset or frustrated
- Finding a way to calm down by getting rid of negative thoughts and emotions and letting positive things into her life.
- There is nothing she can change or go back on. She must learn to accept the facts and move on with a positive attitude.

Evelyn

Resilient Explorer

" I'm calmer now than I used to be. I'm very forgiving of myself."

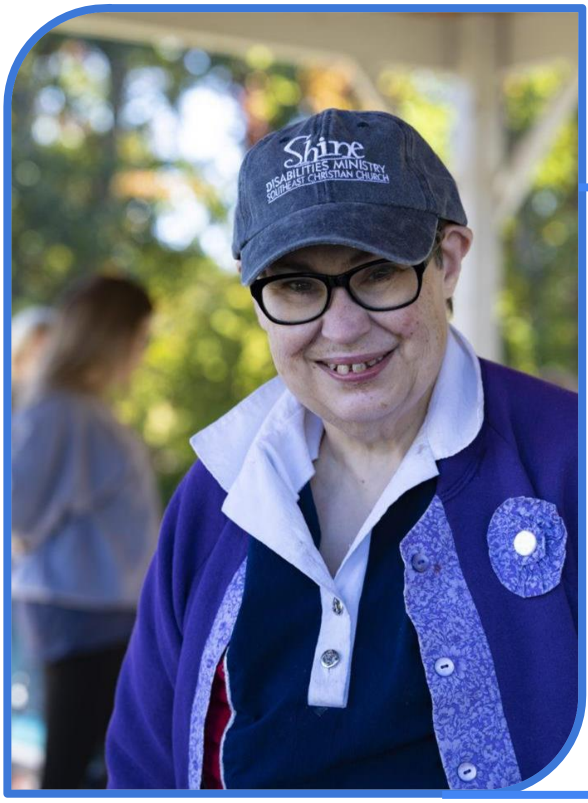

Time Since Injury: 4.5 years
Language: English
Age: 72
Job: Retired (previously involved in community service)
Income: Moderate retirement income
Race: White
Education: Vocational training post high school
Status: Widowed
Living With: Lives alone, but close to family who offer support
Location: Tucson, AZ
TBI Characterization: Significant initial hospitalization, persistent memory issues, slowed cognitive processing, and physical limitations. Experiences challenges with attention ation, emotional regulation, and occasional disorientation. Uses memory aids, personalized strategies, and relies heavily on family support.

GOALS

- Self-compassion and patience with her recovery process.
- Sharing her journey and empathy with others facing similar challenges.
- Finding joy and meaning in daily activities, despite limitations.

BEHAVIOR

- Uses memory aids and associations for daily tasks.
- Adjusts her thoughts and feelings when she suffered from social determinants
- Actively connects with others, showing kindness to those facing physical challenges.
- Maintains calmness and patience in her recovery and daily life.

ATTITUDE

- Accepts her slower pace post-accident, focusing on improvement and self-compassion.
- Forgiving and grateful for progress, especially regaining her personality.
- Empathetic towards those with similar challenges, driven by her own experiences.

MOTIVATION

- Maintains a positive mindset, leveraging gratitude for life as a key motivator.
- Shares compassion and understanding, inspired by personal experiences of adversity.
- Family's supports motivate her to continue her recovery journey and be positive

KEY PERSONALITY ATTRIBUTES

- Self-reliant
- Thankful
- Patient
- Accepting of her recovery journey

BARRIERS

- Slowed physical and cognitive processes due to TBI prevents she from social activities.
- Memory and task completion challenges.
- Feelings of being underestimated by society.

FACILITATORS

- Support and encouragement from family and close friends.
- Memory aids and personalized strategies for managing tasks.
- A strong sense of gratitude and a positive outlook on life, enhancing resilience.

Evelyn

Say:

- When I had first had my accident I was in the hospital for 2 months, and I couldn't. I couldn't even think straight.
- I didn't like like we had to do constant test on. They would ask questions, and I I wanted to avoid them.
- I'm very slow and in in lots of ways, physical ways and thinking ways, and I have to really focus on what I'm doing in order to be able to understand things.
- And so I'm very grateful for that that I am alive and breathing.
- but what I do know is that I feel
- then I'm more compassionate towards people. I feel like I was already compassionate, but I feel like I'm more compassionate because like, for instance, the first time I saw a woman in a wheelchair I I noticed that when I was in a wheelchair and we and when we were in tier we went on a expedition.
- We went to a target, I think, and I noticed that people wouldn't look at me. Avoid me. They would go down a different aisle, cause I was pretty banged up.
- I think just knowing that you need help, and knowing that there's people that can help you. For me. It was family members. They were so encouraging and and also they told me they were gonna kick me out of the hospital if I didn't participate in the tier program that motivated me.

Think

- Physical injury/condition from TBI makes her unable to think straight
- Think her association strategy help her remember things so that she felt better than she suffered from TBI
- Felt grateful of her fate that she is still alive and can breath. Because of the gratification from surviving from TBI, she regards everything happened on her is a good thing.
- She is calmer than before because of survival from TBI
- People avoid her when she was bang up on the wheelchair
- Stoke might be the cause of TBI, because she has both.

Do:

- Used association strategy to help her remember things (i.e. Associating the herbs and garden to her parents' home)
- Focusing on one thing and try best to understand it
- Slow in thinking and physical way
- Felt compassionate after the TBI. Thinking about herself When seeing a woman on the wheelchair. She was treated with indifference by others. Her compassion motivated her to care about others who got hurts.

Feel

- Felt not so much struggled about the current status from TBI since it happened 4.5 years ago
- Felt uncomfortable about being tested constantly (i.e. being asked questions)
- Being forgiving of herself when encountering some problems
- When The bad and good things happened on her, she felt good still because she had her personality back from the TBI
- Grateful of being alive and breath after the accident which make her calmer than used to be.
- Felt being encouraged from her family in TBI recovery
- Felt being treated with indifference by others when she was on the wheelchair
- Skeptical whether TBI was caused by the stroke because she has both
- Encouraging by her families' supports
- Her doctor is good and professional for her TBI treatment

Pains

- Couldn't think straight because of the physical injury/conditions
- Slow in thinking and physical ways. Need to be very focused to understand one thing.
- Being treated with indifference when she was on the wheelchair

Gains

- Having better control on her emotion than she used to be (i.e. be forgiving of herself when she met some problems)
- Association strategy helps her remember things
- Compassionate about others' experience
- Family's supports encourage and motivate her to make her life better

Marcus

Accommodating reflector

"Trying and doing best to get back to my life"

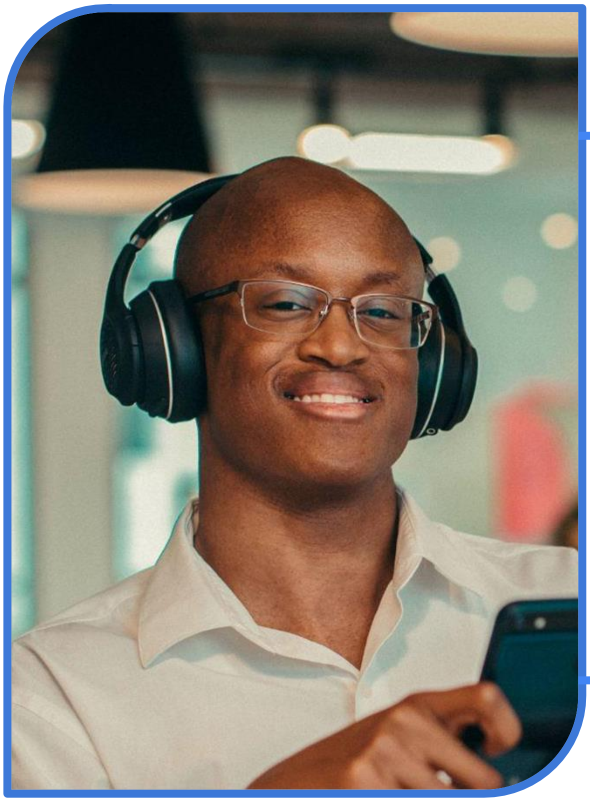

**Time Since Injury**:	8 years
**Language**:	English
**Age**:	35
**Job**:	Freelance photographer
**Income**:	$40,000
**Race**:	Black
**Education**:	Community college (GED)
**Status**:	Married
**Living With**:	Wife, parents and grandmother
**Location**:	Jackson, MS
**TBI Characteristics**:	Academic abilities influenced by pre-existing dyslexia; experiences chronic pain

GOALS

- To be able to regulate his emotions.
- To improve his basic life skills such as walking, getting up, communication, etc.
- To overcome TBI barriers and set a strong example for his children and provide support for his spouse.

BEHAVIOR

- Took "elementary sounding course" to learn how to read again, which was hard but necessary to manage his business.
- Self-checks when he experiences negative emotions to avoid conflicts with his spouse and family members.
- Prefers alternative learning methods, (audiobooks, visual aids) to accommodate his reading and learning difficulties.
- Attends support groups regularly to learn from others' stories and experiences, which helps him improve basic skills and deal with TBI barriers.

ATTITUDE

- Actively reflects on how to calm down and regulate his emotions, helps to maintain a harmonious relationship with his family.
- Willing to learn from others' experiences to cope with his TBI.
- Compassionate about his friend who was in the accident with him; but disappointed he did not also seek medical help after.
- Open to learning from others' experiences and new ways to do things, but often feels insecure about his reading abilities due to his pre-existing dyslexia.

MOTIVATION

- Wants to work on his low level of reading, as it affects his ability to help his children with their homework.
- Encouraged by his grandmother to stay focused on recovery
- Shares his stories to help others; also seeks help from others.

KEY PERSONALITY ATTRIBUTES

- Eager to learn from others
- Accommodating
- High self-esteem
- Polite

BARRIERS

- Challenges with written communication and spelling affecting his ability to write emails, invoices, and social media posts for work.
- Chronic pain affecting his ability to focus on tasks, manage his work effectively, and engage in family activities.
- Reluctant to read due to dyslexia and exhaustion, which makes it hard for him to stay updated with photography trends and technical manuals.

FACILITATORS

- Visual reminders like photos and cards
- Self-check/self-reflection
- Family encouragement

Marcus

**Say**<br>• I had a problem with reading, writing and talking.<br>• I used to try to do everything myself, or I was prone to doing things myself before my accident, but actually, after my accident, you know, it was more relevant that I could do it myself. And I had to come to grips of reality that I couldn't do it no more. I couldn't do it by myself anymore.<br>• It was just a hard test for me to get up and walk through something. you know, without holding a wall, or, you know, come to grips with things of that nature. Like now.<br>• Before I had my accident. I wasn't a very good reader, but I can write and since my accident I have learned how to read and write all over again like sounding out words. I had dyslexia in school.<br>• It's like, you gotta come to group of reality that you're not the same person as you used to be, and your temper. King, and we'll get flared up more easily. You're more prone to getting upset now, and I don't know why is that? It happens you no problem getting upset and taking your emotions out on everybody else.<br>• I I can't speak for everybody, but I don't, can speak for myself.	**Think**<br>• Unwilling to speak and try out learning to speak because kids will tease his reading level.<br>• When come to the reality, he felt he was no longer the same as he used to be<br>• Listening at how other people with similar TBI experience can help him cope with things in his life.<br>• When he can't do something he will take a step back and think about it.<br>• Reflecting the accident resulting in his TBI. He felt sorry about his friend's speaking difficulty and realized the importance of seeking medical helps when got injuries<br>• People's physical conditions are different from what they think after the accidents<br>• He is willing to help people out because he also wants people help him out
**Do**<br>• Went back to learn elementary sounding and read it out.<br>• Did the self-check to reflect on why he screamed at others or couldn't control his feelings<br>• Learning from others' stories how they dealt with their problems and life.<br>• Using the memory tool - a note card can help remind him of the things he wants to buy and way to home<br>• Listened his friend's suggestions that learning how others' read a word and use others' words when speak; Taking photos to memorize how they behave.<br>• Willing to join the program for the recovery to get back to his normal life	**Feel**<br>• Uncomfortable of being teased by kids about his reading level.<br>• Easy to get upset and annoyed<br>• When hearing others stories which are similar to him, he felt chilled.<br>• Helpful from his friend's suggestions that learning from others how they speak and behave<br>• Willing to help fumble persons out using his story.<br>• Positive and hopeful for his life<br>• Motivated by grandmother's words that do and try all things to help you become you and become successful ("do all you can do for yourself")

Pains	Gains
• Difficulty in basic things in life such as getting up, walking through something, reading, etc.<br>• Had dyslexia in school<br>• Easy to get upset and take the emotions out to people around him	• Did the self-check to attempt to regulate the emotion<br>• A card as a memory tool can help remind him of things and way to home in a visual way<br>• Family's support motivate him to do things for himself to get back to normal life.

Lila

Analytical troubleshooter

"Doing my best to have control of myself and get recovered"

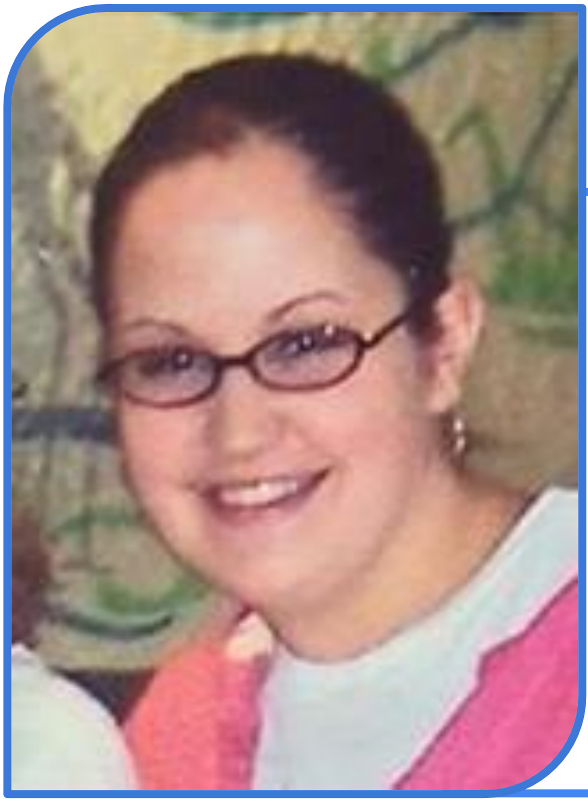

Time Since Injury: 10 years
Language: English
Age: 32
Job: Nurse
Income: $80,000
Race: White
Education: Undergraduate (majored in biochemistry)
Status: Married
Living With: Daughter and husband
Location: Bloomington, IN
TBI Characteristics: Moderate TBI, severe eactions to medications, resulting in memory challenges, aphasia, and emotional regulation difficulties.

GOALS

- To overcome her memory challenges and slight aphasia.
- To be able to control her emotions
- To find more effective strategies to help her recover entirely

BEHAVIOR

- Learned how to be focused and keep track of things during her work
- Uses meditation to help strengthen her memory and improve learning efficiency
- Uses strategies including direct association, repetition and asking for help
- Regulates her emotions by telling herself that being mad doesn't fix anything

ATTITUDE

- Determined to work hard to be successful
- Hopeful to overcome TBI barriers and sets goals for this
- Believes that others' stories and strategies can help her cope with TBI challenges
- Places trust in the authority of medical experts and scientific treatment and learning materials
- Frustrated when she couldn't handle the things she could do before TBI

MOTIVATION

- Listening to others' TBI stories can help shape her own TBI story.
- The professional experience of medical experts.
- Learning better strategies to cope with her TBI at work.

KEY PERSONALITY ATTRIBUTES

- Insightful
- Confident
- Self-regulated
- Scientifically-minded

BARRIERS

- Difficulty remembering things and sometimes speaking
- Difficulty regulating her emotions
- Hard to accept that she can't do what she used to do
- Medication can result in severe cardiac problems for her

FACILITATORS

- Professional therapy from experts
- Others' stories
- Useful strategies to cope with TBI
- Work experience

Lila

**Say**	• My car accident was 10 years ago, and so I've greatly recovered in that time. Thing the good Lord above.<br>• I would say initially, one of the really tough things was retention, retaining things and memory, so that those are the those are the really big problems that I dealt with. Retaining things in memory and then focus to and focus. I think definitely is still an issue that I have.<br>• And I would spend, you know, all day weeks on in preparing for exams, and then go to sleep, and the next day wake up, and it was like I never studied anything and so I at the time was taking<br>• a medication to kind of help with that, and then I had to stop taking the medication because my heart had been I was resuscitated 4 times, and so my heart had been decommissioned is what the doctor called it.<br>• But, like you said, too, I had a lot of aphasia I was having, I still I still, and it still frustrates me a lot. I still have trouble finding words. I still have that expressive aphasia sometimes, and it it's very frustrating.<br>• Cause I I do get really mad sometimes really frustrated.<br>• All your memory already stinks because of the brain injury, and then your memory also makes it harder to learn things to overcome the memory problem.	**Think**	• Retaining things in memory is still an issue for her<br>• Had to stop the medication since it will cause the cardiac problems<br>• Her memory problems made her fail the exams instead of her lack of preparation<br>• Willing to spend time to find and try out strategy to help her remember things and speak.<br>• Encourage herself to overcome TBI barriers and accept the fact of TBI and decide to manage it<br>• Situational awareness on her brain efficiency and emotions are her big problems<br>• Be positive about the effect of strategy that can help the TBI<br>• Listening to other survivors' stories can help other to recover<br>• Sharing the stories can give patients and their caregivers' hopes<br>• Repetition is a good strategy to make people's learning easier<br>• Repetition and knowing when and how to ask helps can be helpful for individuals with TBI<br>• Believed that people with lived experience can provide great insights<br>• It will be better to make or entice the patient to seek help and think they are in their controls<br>• The statistic evidence of a medical program is persuasive
**Do**	• Learn how to focus and keep track of things during her work<br>• Studies hard to pass the exams and used the medication to improve learning efficiency<br>• Used the direct association strategy to help her remember things<br>• Used the "keep things in order" strategy to help her clarity<br>• Regulating her emotions by telling herself that being mad cannot fix anything<br>• Try to implement the similar strategies that has helped others	**Feel**	• The accident happened in 2014 still distinguished in her memory<br>• Acceptable about TBI on her and be active and confident to manage it.<br>• Unreasonable to be mad since it cannot fix anything<br>• Struggled with the memory phase in the recovery journey<br>• It is easy for people to repeat behaviors which seems like the routines in life that people don;t need to think about how and why they need to do that<br>• Trustworthy about the medical experts' treatment and learning materials

Pains	Gains
• Hard to retention and focus and remember things<br>• Failed the exams because of her memory<br>• The medication will help her recovery but it will cause cardiac problems<br>• Expressive aphasia<br>• Unable to control her emotions	• Learned to focus and keep track of things from her work<br>• Used the association strategy to support her memory<br>• Find the layman terms in open resources to help her enhance clarity<br>• Others' stories help her build a successful story<br>• Learned practical skills of dealing with TBI patients

Carlos

Resilient practitioner

"Kicking out all the barriers. I can manage it"

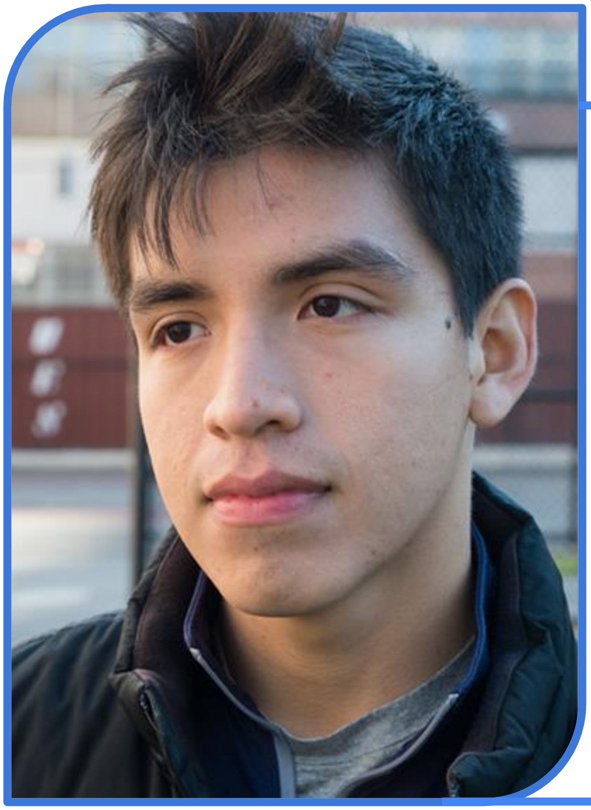

Time Since Injury: 1 year
Language: English, Spanish
Age: 19
Job: Student, athlete (soccer)
Income: 25 dollars/ hr
Race: Latino
Education: Second-year undergraduate
Status: Single
Living With: Parents and a puppy
Location: Los Angeles, CA
TBI Characteristics: Memory loss, difficulty regulating behaviors and emotions, slowed learning processes, chronic headaches, fatigue, and sensitivity to noise and light. Relies heavily on physical therapy, expert guidance, and family support.

GOALS

- Getting back to professional athlete career.
- To be able to control his behaviors and emotions.
- To not be treated differently by his peers.

BEHAVIOR

- Continuing his college classes, but feels exhausted often after class.
- Uses notes and flashcards to remember things and learn, but needs rest after learning.
- Working with experts for TBI treatment.
- Uses physical practice and rehabilitation as an outlet.
- Seeks help from his family.
- Often vents nonymously on social media

ATTITUDE

- Trusts the help from experts and family
- Believes physical therapy is scientific and professional and will help him
- Accepts the reality of his TBI, but is determined to make changes and recover
- Masks his TBI; feels if others know, he may not be able to play soccer at college

MOTIVATION

- Does not want to lose his soccer career
- Doesn't want to let his parents down
- Wants to have a strong body again

KEY PERSONALITY ATTRIBUTES

- Introverted
- Obedient
- Seems older than he is
- Caring

BARRIERS

- Difficulty remembering things
- Difficulty regulating her behaviors and emotions
- Very slow to learn new things post injury
- Old friends avoid him and teammates treat him differently

FACILITATORS

- Taking notes, flash cards
- Family support
- An emotional support animal
- Soccer career
- Physical workout to relieve the stress

Photo credits:

Jordan photo: Retrieved 3/13/2024 from: https://unsplash.com/photos/woman-in-white-tank-top-UIrgThPxuJ4 ; Free to use under the Unsplash License

Casey photo: Retrieved 3/22/2024 from: https://unsplash.com/photos/woman-on-focus-photography--bMJU-lPI ; Free to use under the Unsplash License

Riley photo: Retrieved 6/4/2024 from: https://unsplash.com/photos/a-man-pushing-a-suitcase-down-a-sidewalk-pZQFJjVS3Fg ; Free to use under the Unsplash License

Morgan photo: Retrieved 3/27/2024 from: https://unsplash.com/photos/woman-standing-front-of-vine-plant-bsU_7Ln2E2Y ; Free to use under the Unsplash License

Lila photo: Retrieved 4/9/2024 from https://www.flickr.com/photos/kstrandlund/19185606735/ ; License: CC BY-NC 2.0 DEED; Attribution-NonCommercial 2.0 Generic

Carlos photo: Retrieved 4/9/2024 from: https://www.flickr.com/photos/danielfoster/38395447066 ; License: CC BY-NC 2.0 DEED Attribution-NonCommercial 2.0 Generic

Short Bios of the Eleven "Personas"

Alexis – Balanced Contemplator

Alexis is a 42-year-old woman who lives with her family in the city. She loves reading, learning new things, and spending quiet time thinking. Alexis has a college degree and works part-time at a local community center where she helps others feel supported and included. Alexis is a proud Black woman and a survivor of a TBI she experienced in a car accident 8 years ago where she lost consciousness for over 30 minutes but less than one day. She continues to deal with challenges like memory problems and mental fatigue, but she has learned to use strategies to manage these day by day so she can continue to engage in activities that are meaningful to her. Alexis reminds everyone that their injury does not define them.

Jordan – Resilient Maverick

Jordan is a 27-year-old woman who works as an artist and lives with her partner and two cats. She is proud of her cultural roots as a Latina and uses her creativity to express herself in her art and her community work. Jordan survived a brain injury from a sports accident when she was 19; she did not lose consciousness but doesn't remember the injury very clearly. Though her injury was called 'mild,' she faced real challenges with focus, energy, and managing emotions. Jordan believes her lived experiences, including her disability, are her strengths. She loves helping others see the power in their own story.

Taylor – Resourceful Innovator

Taylor is a 51-year-old white man who loves being outdoors, building things with his hands, and volunteering in his community. He works part-time at a local hardware store and is known for his kindness and sense of humor. Taylor experienced a severe brain injury over 20 years ago after falling from a ladder while working. He lives with some physical challenges, including balance and speech difficulties, but he is always looking for new ways to adapt and succeed. Taylor shows others that even when life changes in big ways, new opportunities can be found.

Casey – Adaptive Veteran

Casey is a 49-year-old Latina woman who served in the military before starting a career in digital marketing. She is a proud mother of two and loves cooking and spending time with her family. Casey experienced a brain injury during her service 15 years ago. Since then, she has worked hard to adjust to changes in attention, memory, and the fast pace of digital work. Casey helps others learn how to balance old traditions with new ways of doing things, reminding people that change can be a good thing.

Riley – Accommodating Empath

Riley is a 64-year-old white man who lives in a small town with his wife. He enjoys woodworking and being active in his local church. Riley fell and hit his head after having a stroke 6 years ago. Since then, he has worked on improving his patience, thinking more before speaking or acting, and adjusting to changes in his energy levels. Riley believes that

slowing down and being thoughtful is a strength, and he helps others feel understood and supported.

Morgan – Positive Stabilizer

Morgan is a 32-year-old Black woman who works in customer service and lives with her two dogs. She loves nature, music, and helping people. Morgan had a brain injury in a car crash 5 years ago. Since then, she has worked hard to rebuild her confidence and take steps toward the life she wants. Morgan faces challenges with organization and emotional ups and downs, but she keeps moving forward. She reminds others that it's okay to work hard for the life you deserve and to find joy in the little things.

Evelyn – Resilient Explorer

Evelyn is a 58-year-old white transgender woman who enjoys traveling, exploring parks, and spending time with friends. She is retired and spends her time volunteering at a local library. Evelyn had a brain injury more than 30 years ago in a car accident. Over the years, she has learned how to manage frustration, stay calm, and be kind to herself when things don't go as planned. Evelyn's journey shows that it's never too late to learn new things and keep exploring.

Marcus – Accommodating Reflector

Marcus is a 38-year-old Black man who works in tech support. He enjoys music, gaming, and hanging out with friends. Marcus experienced a brain injury 10 years ago after a fall; he lost consciousness for 2 hours. Since then, he has been working to get back to the life he loves. Marcus sometimes struggles with focus and energy, but he keeps trying every day. He encourages others to be patient with themselves and to celebrate every small success along the way.

Lila – Analytical Troubleshooter

Lila is a 29-year-old white woman who works as a data entry specialist. She enjoys puzzles, board games, and spending time with her siblings. Lila had a mild brain injury after a bike accident when she was 17. Even though her injury seemed small at the time, she struggled with controlling her emotions and staying organized after. Lila uses her problem-solving skills to stay on track and help others do the same. She shows that thinking things through can make a big difference in recovery.

Carlos – Resilient Practitioner

Carlos is a 23-year-old Latino man who is finishing college while working part-time in a coffee shop. He loves sports, hanging out with friends, and trying new things. Carlos had a brain injury during a soccer game in high school. He faced barriers like being told what he couldn't do, but Carlos works hard every day to prove to himself and others that he can manage it. He encourages everyone to keep kicking down the barriers that try to stand in their way.
