# Supplementary material for: Designing Electronic Problem-Solving Training for Individuals With Traumatic Brain Injury: Mixed Methods, Community-Based, Participatory Research Case Study
Source: J Med Internet Res. 2026 Jan 20;28:e83995. doi: 10.2196/83995 (PMC12818508; doi:10.2196/83995)
Supplement: Checklist 1 [file jmir-v28-e83995-s002.pdf]

GRIPP 2 Short Form

Section and topic	Item	Reported on page No
1: Aim	Report the aim of PPI in the study	12
2: Methods	Provide a clear description of the methods used for PPI in the study	13-18
3: Study results	Outcomes—Report the results of PPI in the study, including both positive and negative outcomes	19-32
4: Discussion and conclusions	Outcomes—Comment on the extent to which PPI influenced the study overall. Describe positive and negative effects	33-38
5: Reflections/critical perspective	Comment critically on the study, reflecting on the things that went well and those that did not, so others can learn from this experience	38-39
